# RARS1 inhibits ENO1 ubiquitination and degradation to protect against ferroptosis in hepatocellular carcinoma

**DOI:** 10.3389/fimmu.2025.1686597

**Published:** 2025-12-10

**Authors:** Shouge Zang, Jvlong Ma, Lichang Chen, Di Cui, Jiangtao Yu

**Affiliations:** 1 Department of Hepatopancreatobiliary Surgery, Fuyang People’s Hospital of Anhui Medical University, Fuyang, Anhui, China; 2 Clinical Laboratory, No.2 People’s Hospital of Fuyang City, Fuyang, Anhui, China; 3 Fuyang Medical College, Fuyang Normal University, Fuyang, Anhui, China

**Keywords:** arginyl-tRNA synthetase, ENO1, allograft rejection, ubiquitination, ferroptosis

## Abstract

**Background:**

Liver hepatocellular carcinoma (LIHC) is an aggressive malignancy with high recurrence and therapy resistance. Transplant rejection-related genes (ARRGs) have emerged as potential contributors to cancer progression. This study investigates the role of RARS1, a gene involved in protein synthesis, in LIHC progression and its therapeutic potential.

**Methods:**

AR-DEGs in LIHC were identified via differential expression and Cox regression analyses, followed by non-negative matrix factorization (NMF) to classify patients into molecular subtypes. Immune microenvironment, immune evasion, and stemness differences were assessed. Multi-omics datasets, including transcriptomic, single-cell, and spatial transcriptomics, were used to evaluate RARS1 expression. Bioinformatics and molecular biology techniques were employed to study RARS1’s role in oncogenic pathways, immune modulation, and ferroptosis, including interaction with ENO1. Drug sensitivity analysis identified potential RARS1-targeting compounds.

**Results:**

Three LIHC subtypes with distinct immune landscapes and prognoses were identified. Cluster 1 exhibited high immune infiltration and poor response to immune checkpoint blockade. RARS1 was significantly overexpressed in LIHC and correlated with poor prognosis. Knockdown of RARS1 inhibited proliferation and migration of LIHC cells and influenced immune cell polarization. Mechanistically, RARS1 regulated the PI3K/AKT/GSK3β pathway and suppressed ferroptosis via ENO1. Drug analysis revealed AH.6809 as a potential inhibitor of RARS1, reducing its oncogenic effects *in vitro*.

**Conclusion:**

AR-DEG-based subtyping reveals distinct LIHC immune profiles. RARS1 promotes LIHC progression through oncogenic signaling and immune modulation, serving as a promising prognostic biomarker and therapeutic target. Targeting RARS1 with agents like AH.6809 may offer novel treatment strategies for LIHC.

## Introduction

Liver hepatocellular carcinoma (LIHC) is a highly prevalent and aggressive cancer with significant morbidity and mortality, posing a major public health threat, particularly in individuals with chronic liver diseases such as hepatitis and cirrhosis (1). Despite recent advances in early diagnosis and treatment, LIHC remains a major therapeutic challenge due to its high recurrence rate and resistance to conventional therapies (2).

Liver transplantation (LT) has become a well-established and highly effective treatment for patients with hepatocellular carcinoma who meet specific criteria, such as having a limited tumor burden and being unsuitable for surgical resection (3). However, even among patients who fulfill the strict Milan criteria for LT, the recurrence risk of LIHC after 5 years remains between 10% and 15% (4). This recurrence is often driven by complex immune processes, including allograft rejection, which involve multiple immune cells and signaling pathways. Long-term use of immunosuppressive agents is essential to prevent organ rejection, but excessive immunosuppression can impair immune surveillance, increasing the risk of tumor recurrence and metastasis (5). On the other hand, insufficient immunosuppression may cause rejection of the graft (6). Thus, accurately assessing and balancing immune status is crucial to reducing the risk of postoperative tumor recurrence.

Recent study has revealed an unexpected overlap between the mechanisms of transplant rejection and cancer immunotherapy (7), suggesting that genes related to allograft rejection-related genes (ARRGs) not only play pivotal roles in transplant rejection but may also contribute to the initiation and progression of tumors. In this context, we utilized bioinformatics analysis to extract expression and clinical data from the TCGA database to identify genes associated with transplant rejection in LIHC. We performed unsupervised clustering analysis on the expression patterns of ARRGs in LIHC samples, leading to the identification of distinct molecular subtypes within the disease.

Arginine tRNA synthetase 1 (RARS1), located on chromosome 5, encodes a 641-amino acid protein that is critical for maintaining cellular physiological functions, particularly in protein synthesis (8). Recent studies have increasingly highlighted the involvement of RARS1 in several malignant tumors, demonstrating its association with key oncogenic processes such as cell proliferation, apoptosis, and metabolic reprogramming (9, 10). Despite these findings, the expression profile and biological functions of RARS1 in LIHC remain largely unexplored. In this study, we integrated multi-omics data with molecular biology approaches to investigate the differential expression of RARS1 in LIHC and to explore its potential mechanisms of action. Our results provide an experimental foundation for further exploration of RARS1 as a promising therapeutic target in LIHC.

## Materials and methods

### Data acquisition

Gene transcriptome and clinical data, including gender, age, race, pathological stage, alpha-fetoprotein (AFP) levels, radiotherapy, chemotherapy status, and survival information, were obtained from The Cancer Genome Atlas (TCGA) (https://www.cancer.gov) for liver hepatocellular carcinoma (LIHC) tumor tissue samples (n = 374) and normal liver tissue samples (n = 50). The allograft rejection-related genes (ARRGs) were retrieved from the MSigDB database, comprising a total of 200 genes. Single-cell RNA sequencing (scRNA-seq) data were acquired from the Tumor Immune Single-cell Hub (TISCH) database (GSE166635), which includes two LIHC samples. Spatial transcriptomics data were obtained from the Gene Expression Omnibus (GEO) (GSM6177612), containing 1 LIHC tissue sample.

### Acquisition of LIHC samples

Between January 1, 2022, and December 31, 2023, LIHC patients admitted to Fuyang People’s Hospital of Anhui Medical University, were enrolled in this study. Tissue samples were collected from 48 patients undergoing curative resection. Among them, eight pairs were used for RT-PCR and Western blot analyses, while the remaining 40 pairs were outsourced to Qingdao Spaiter Biotechnology Co., Ltd. for tissue microarray preparation and immunohistochemical staining. Freshly excised samples were rinsed with saline and immediately preserved in liquid nitrogen. All samples were pathologically confirmed as LIHC, and the study protocol was approved by the Ethics Committee of Fuyang People’s Hospital.

### Differential genes and prognostic genes analysis

Differential expression analysis of ARRGs in LIHC tumor versus normal tissues was performed using the “limma” package in R, with thresholds set at an adjusted false discovery rate (FDR) < 0.05 and absolute log2 fold change (|log2FC|) ≥ 0.5. Prognostic gene screening was conducted using the “survival” package, employing Cox proportional hazards regression to assess the relationship between gene expression and overall survival (OS). Prognostic genes were selected with a significance threshold of *p* < 0.05. The intersection of differentially expressed ARRGs and prognostic genes was defined as allograft rejection-related differentially expressed genes (AR-DEGs).

### Clustering analysis

Non-negative matrix factorization (NMF) was applied to cluster LIHC tumor samples based on AR-DEG expression, constructing molecular subtypes of LIHC associated with antigen recognition resistance. Prognosis among subtypes was compared, and tumor microenvironment differences were explored. Immune cell infiltration was evaluated using the “immunedecon” package with the TIMER algorithm. Expression levels of immune checkpoint-related genes (ITPRIPL1, SIGLEC15, TIGIT, CD274, HAVCR2, PDCD1, CTLA4, LAG3, and PDCD1LG2) were extracted from TCGA datasets (11–13). The TIDE algorithm was employed to assess immune evasion mechanisms and predict immunotherapy response, with higher TIDE scores indicating poorer responses to immune checkpoint blockade (ICB) therapy and shorter survival. The progression of cancer typically involves a gradual loss of differentiated phenotypes and the acquisition of stem-like features resembling progenitor or stem cells (14). To assess the stemness of the samples, we employed the OCLR algorithm developed by Malta et al (15). This algorithm calculates a stemness index based on transcriptomic data from the samples, allowing for the evaluation of stemness differences across various subtypes of tumor cells.

### Clinical correlation and prognostic analysis

Patients were divided into high- and low-expression groups based on the median expression level of RARS1. The association between RARS1 expression and clinical features, including TNM stage and pathological grade, was analyzed. Kaplan-Meier survival analysis was conducted to evaluate the correlation between RARS1 expression and patient survival, with statistical significance determined by the log-rank test. Immune infiltration analysis of RARS1 in LIHC was performed using the “GSVA” package with Spearman correlation analysis.

### Single-cell data analysis

Gene expression at single-cell resolution was obtained from the TISCH database. The “Seurat” package was used for data normalization and identification of highly variable genes. Principal component analysis (PCA) and clustering analysis were performed to classify different cell subpopulations. The Kruskal-Wallis test was used to assess differential RARS1 expression across cell types. Cells were classified into RARS1-positive and RARS1-negative groups, and the proportion of each cell type in both groups was calculated.

### Cell-cell communication analysis

Cell-cell communication analysis was conducted using the “CellChat” package, predicting intercellular signaling based on known ligand-receptor interactions. The resulting network was visualized using the “ggplot2” package, where nodes represent cell types and edge thickness indicates communication strength.

### Spatial transcriptomics analysis

To assess cellular composition at each spatial transcriptomics spot, deconvolution analysis was performed. A reference scRNA-seq dataset was constructed using scRNA-seq data from multiple LIHC samples. Stringent quality control was applied based on gene expression, unique molecular identifier (UMI) counts, and mitochondrial RNA percentage. A signature score matrix was generated by averaging the top 25 marker genes per cell type, followed by enrichment analysis using “Cottrazm”. Spatial distribution of cell types was visualized with “SpatialFeaturePlot” in “Seurat”. Spearman correlation analysis was performed using the “linkET” package to examine relationships between cell composition and gene expression.

### RNA extraction and quantitative real-time PCR

Tissue samples were homogenized with TRIzol reagent, and RNA was extracted using chloroform, isopropanol precipitation, and 75% ethanol washing. RNA concentration and purity were measured using a NanoDrop spectrophotometer. cDNA synthesis was performed with a reverse transcription kit, and gene expression was quantified using SYBR Green qPCR Master Mix. Relative gene expression was calculated using the ΔΔCt method with ACTB as the internal control. Results are presented as mean ± SD, with statistical significance. The primers used in this study are listed in **Supplementary Table 1**.

### Western blotting

Proteins were extracted using RIPA buffer with protease inhibitors, and protein concentration was measured by the BCA assay. Proteins were separated by SDS-PAGE, transferred to PVDF membranes, and blocked with 5% skim milk. Membranes were incubated with primary antibodies overnight, followed by HRP-conjugated secondary antibodies. Protein expressions were detected using ECL and quantified using ImageJ software. The antibodies used included RARS1(Proteintech, China), ENO1(Biyuntian, China), PI3K(Proteintech, China), AKT(Proteintech, China), p-AKT(Proteintech, China), GSK3β(Proteintech, China) and β-Actin (Proteintech, China).

### Immunohistochemical staining

Tissue sections (4µm) from formalin-fixed, paraffin-embedded samples were deparaffinized in xylene and rehydrated through a graded ethanol series. Antigen retrieval was conducted with citrate buffer, followed by blocking of endogenous peroxidase with 3% hydrogen peroxide. Sections were incubated overnight at 4 °C with primary antibodies, and after washing, secondary antibody incubation was performed. Visualization was achieved using DAB, with nuclei counterstained with hematoxylin. Slides were dehydrated, cleared, and mounted for examination. Immunohistochemical scoring was performed by two experienced pathologists.

### Cell culture

The LIHC cell lines MIHA, SKHep1, PLC, HepG2, and Huh7 were obtained from Wuhan Servicebio Co., Ltd. Cells were cultured in DMEM supplemented with 10% fetal bovine serum (FBS) and 1% penicillin-streptomycin. Cells were maintained in a 37 °C incubator with 5% CO2. Media were changed every 2–3 days, and cells were passaged when they reached 80% confluence.

### Cell migration assay

For the migration assay, cells were placed in the upper chamber of Transwell plates with 8-µm pores, using serum-free medium. The lower chamber contained DMEM supplemented with 20% FBS. After 36–48 hours of incubation, the cells on the membrane were fixed using 4% paraformaldehyde and then stained with 0.5% crystal violet. Migrating cells were observed and counted under a microscope.

### Cell scratch assay

Cells were grown to full confluence in 6-well plates, and a sterile pipette tip was used to create a scratch. After removing floating cells, the cultures were maintained in serum-free medium for 24 hours. The gap closure was monitored and photographed at 0 and 24 hours to assess cell migration.

### Colony formation assay

Cells were seeded in 6-well plates at a density of 200 cells per well and cultured in complete medium for 10 to 14 days. After colony formation was visible, the cells were fixed with methanol and stained with crystal violet. The colonies were observed under a microscope, and the number of colonies was counted.

### Cell cycle analysis

Cells in the logarithmic phase of growth were fixed in 70% ethanol at -4 °C overnight. After washing with PBS, cells were stained with propidium iodide (PI) for 30 minutes and analyzed using flow cytometry. The cell cycle distribution was analyzed with ModFit software.

### Transfection and stable cell line construction

For transient transfection, cells were grown to 80% confluence, and Lipofectamine 8000 transfection reagent was used to deliver plasmid or siRNA constructs. After 16 hours, the medium was replaced with fresh growth medium. Cells were harvested for 48 hours post-transfection for subsequent analysis. Stable knockdown cell lines were generated using lentiviral-mediated shRNA targeting RARS1. shRARS1 and the negative control shRNA were cloned into the SV40-Puro lentiviral vector. Lentivirus was produced in 293T cells, and target cells were infected with viral particles and selected with puromycin. Knockdown efficiency was validated by qRT-PCR and Western blot.

### RNA sequencing

To investigate the transcriptomic characteristics of SKHep1 cells, we performed RNA-seq on amplified samples from both the control and knockdown groups. The sequencing was conducted by TsingkeBiotechnologyCo., Ltd. Raw reads were first filtered using Trimmomatic to remove low-quality sequences. After quality control, STAR was employed for alignment. Differentially expressed genes (DEGs) were identified, annotated, and subjected to clustering analysis. KEGG pathway enrichment analyses were performed to explore potential biological functions and pathways.

### Co-immunoprecipitation

Cells were lysed with RIPA buffer, and the supernatant was incubated with agarose beads and RARS1 antibody overnight at 4 °C. After washing, the beads were resuspended in SDS loading buffer and boiled for 10 minutes. The eluted proteins were separated by SDS-PAGE and identified by mass spectrometry.

### Ubiquitination assay

Cells were treated with the proteasome inhibitor MG132 for 24 hours to block protein degradation. Cell lysates were incubated with anti-ubiquitin (PTM, China) or target protein antibodies for immunoprecipitation. Ubiquitinated proteins were captured using protein A/G magnetic beads and analyzed by Western blot.

### HPLC analysis

HPLC was used to analyze protein complexes that interact with RARS1. Protein extracts were immunoprecipitated with RARS1 antibody and eluted proteins concentrated. Proteins were separated by reverse-phase C18 column chromatography with a linear acetonitrile gradient and detected at 214 nm and 280 nm.

### Immunofluorescence

Cells were fixed with 4% paraformaldehyde and permeabilized using 0.1% Triton X-100. After blocking with goat serum, they were incubated with primary antibodies overnight at 4 °C. The cells were then stained with fluorophore-conjugated secondary antibodies and DAPI for nuclear labeling. Fluorescent images were captured using a confocal microscope, and protein localization was analyzed with ImageJ software.

### ROS detection

ROS levels were assessed using the DCFH-DA probe (Biyuntian, China). Cells were incubated with 10 μM DCFH-DA for 20 minutes at 37 °C, then washed with serum-free medium. ROS generation was visualized using a confocal microscope.

### Scanning electron microscopy

Cells were fixed in 2.5% glutaraldehyde for 24 hours, washed with PBS, and post-fixed with 1% osmium tetroxide for 2 hours. After dehydration in ethanol, critical point drying, and gold sputtering, SEM imaging was conducted using a JEM-1400 electron microscope.

### THP-1 polarization and induction

THP-1 cells were treated with PMA (100 ng/mL) for 24 h and then cultured in fresh complete medium for another 24 h to obtain M0 macrophages. The M0 cells were randomly divided into two parts: one part was directly incubated with tumor cell–conditioned medium (TCM) derived from NC or Sh1 cells for 24–48 h (M0 + TCM groups); the other part was stimulated with IL-4 (20 ng/mL) and IL-13 (20 ng/mL) for 24 h to induce M2 macrophages, followed by replacement with the corresponding TCM (M2 + TCM groups). The polarization status was evaluated by flow cytometry (CD206 and CD86), and cytokine expression levels were assessed by qPCR for ARG1, IL10, IL12A, and TNF.

### Animal studies

BALB/c nude mice (6-week-old females) were randomly divided into four groups (n=4/group). Stable cell lines (5×10^6^ cells in 200μL PBS) were injected subcutaneously into the right flank. Tumor growth was monitored with calipers, and tumor volumes were calculated as: Volume = 1/2 × length × width². Tumor weight, morphology, and Ki67 expression were assessed by immunohistochemical staining.

### Drug sensitivity analysis

RARS1 drug sensitivity was analyzed using the GSCA database (http://bioinfo.life.hust.edu.cn/GSCA/#/drug). Additionally, a Connectivity Map (cMAP) analysis was conducted to identify potential therapeutic compounds (16). Using the XSum method, we compared gene features with cMAP signatures, calculating similarity scores for 1288 compounds. Low similarity scores indicated potential inhibitors of RARS1-mediated oncogenesis.

### Statistical analysis

Statistical analyses were performed using R 4.2.1, and visualizations were created with the “ggplot2” package. Group comparisons were conducted using t-tests or Mann-Whitney U tests, with *p* < 0.05 considered statistically significant.

## Results

### Screening of AR-DEGs and cluster analysis

**Figure 1:**
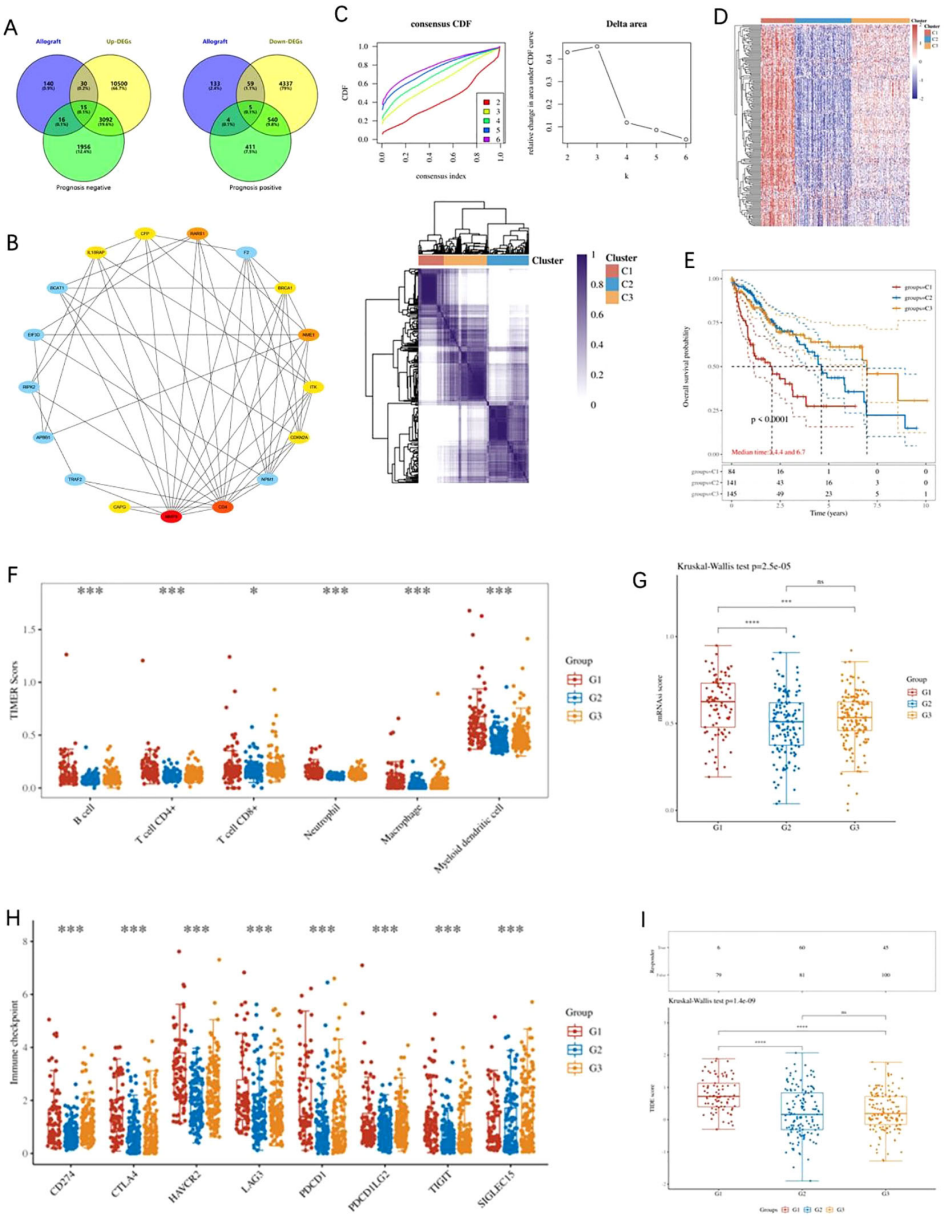
Analysis of AR-DEGs in LIHC. **(A)** Venn diagram depicting the overlap between differentially expressed genes (DEGs), resistance-related genes, and prognosis-related genes, highlighting 15 upregulated and 5 downregulated genes. **(B)** Protein-protein interaction (PPI) network of AR-DEGs. The network was constructed based on the MCC algorithm, identifying 10 key hub genes, including IL18RAP, CFP, RARS1, BRCA1, NME1, ITK, CDKN2A, CD4, MMP9, and CAPG. **(C)** K-means consensus clustering analysis of 374 LIHC patients from TCGA based on the expression of 10 AR-DEGs. Consensus clustering curves (left) and delta area plot (right) are shown, with the patients classified into two distinct clusters. **(D)** Heatmap showing the expression patterns of AR-DEGs across the identified subgroups, highlighting significant differences in gene expression between the clusters. **(E)** Kaplan-Meier survival curves demonstrate the worse prognosis for patients in Cluster 1 compared to those in Clusters 2 and 3. **(F)** Immune cell infiltration analysis using the TIMER algorithm, revealing significantly higher immune cell infiltration in Cluster 1 compared to other clusters across six immune cell types. **(G)** Tumor stemness scoring indicates that Cluster 1 patients exhibit higher stemness properties compared to other subgroups. **(H)** Expression analysis of immune checkpoint-related genes (TPRIPL1, TIGIT, CD274, HAVCR2, PDCD1, CTLA4, LAG3, and PDCD1LG2), which are significantly upregulated in Cluster 1. **(I)** TIDE analysis indicating that Cluster 1 patients have a poorer response to immune checkpoint blockade (ICB) therapy. Data were shown as mean ± SD. *p < 0.05, **p < 0.01, ***p < 0.001, ****p < 0.0001.

Utilizing the VennDiagram package, we conducted an intersection analysis of ARGs, differentially expressed genes (DEGs), and prognosis genes, identifying 15 upregulated and 5 downregulated genes (**Figure 1A**). Based on protein-protein interaction (PPI) network data, we employed the MCC algorithm to identify 10 AR-DEGs within the network: IL18RAP, CFP, RARS1, BRCA1, NME1, ITK, CDKN2A, CD4, MMP9, and CAPG (**Figure 1B**). Subsequently, we performed K-means consensus clustering analysis on 374 LIHC patients from TCGA using these 10 AR-DEGs. Based on the area under the consensus clustering curve and k-value, the patients were classified into two clusters (**Figure 1C**). Expression heatmaps revealed distinct gene expression patterns between subgroups, providing a foundation for further exploration of their biological functions (**Figure 1D**). Kaplan-Meier survival analysis showed that patients in Cluster 1 had significantly worse prognosis compared to Clusters 2 and 3 (**Figure 1E**). Using the TIMER algorithm, we evaluated immune cell infiltration across molecular subtypes, revealing that Cluster 1 exhibited higher infiltration levels across six immune cell types (**Figure 1F**). Tumor stemness scoring further indicated that Cluster 1 patients had higher stemness properties (**Figure 1G**). Additionally, immune checkpoint-related genes, including TPRIPL1, TIGIT, CD274, HAVCR2, PDCD1, CTLA4, LAG3, and PDCD1LG2, were significantly upregulated in Cluster 1 (**Figure 1H**). TIDE analysis suggested that Cluster 1 patients exhibited poorer response to immune checkpoint blockade (ICB) therapy (**Figure 1I**). These results suggest that AR-DEGs effectively classify LIHC patients, with Cluster 1 displaying higher immune infiltration, worse therapeutic response, and poorer prognosis.

### Expression, prognostic significance, and clinical features of RARS1 in LIHC

**Figure 2:**
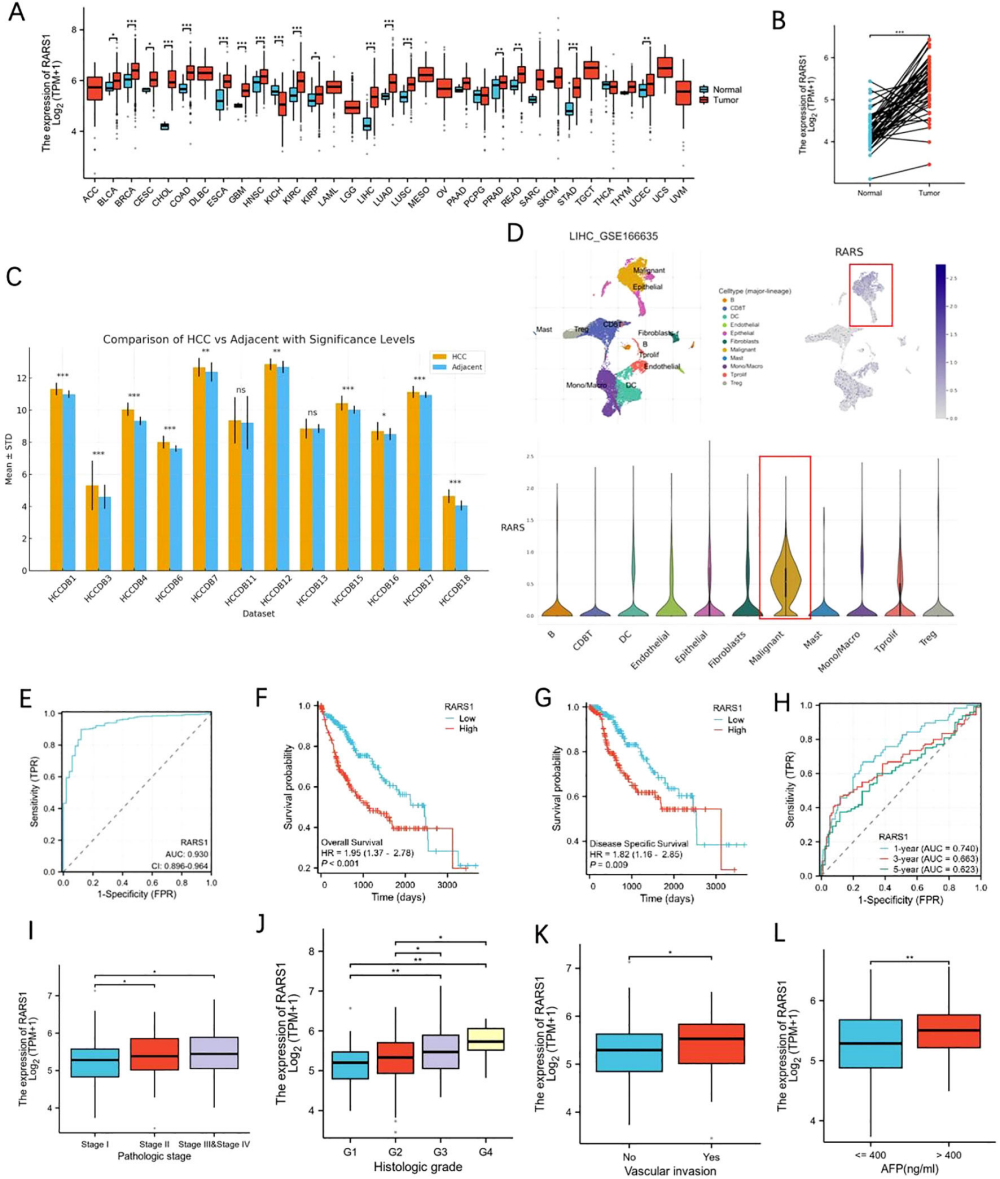
Expression and prognostic significance of RARS1 in LIHC. **(A)** Pan-cancer analysis of RARS1 expression across 17 different cancer types, including LIHC, using TCGA datasets, showing significant upregulation of RARS1 in various cancers. **(B)** Paired sample analysis confirming that RARS1 expression is significantly elevated in LIHC tumor tissues compared to adjacent normal tissues. **(C)** Validation of RARS1 upregulation across multiple LIHC datasets, further confirming its elevated expression in tumor samples. **(D)** Single-cell RNA sequencing analysis showing that RARS1 is predominantly expressed in malignant tumor cells within the tumor microenvironment. **(E)** Receiver Operating Characteristic (ROC) curve analysis of RARS1, demonstrating its strong diagnostic performance for LIHC with an AUC of 0.924. **(F)** Kaplan-Meier survival analysis revealing that patients with high RARS1 expression have significantly poorer overall survival (OS) compared to those with low expression. **(G)** Disease-specific survival (DSS) analysis showing that high RARS1 expression is associated with a worse DSS. **(H)** ROC curve analysis further confirming that RARS1 effectively predicts 1-, 3-, and 5-year survival rates in LIHC patients. **(I)** Box plot illustrates the correlation between RARS1 expression and pathological stage in LIHC patients. **(J)** Box plot showing the association between RARS1 expression and histologic grade in LIHC. **(K)** Box plot demonstrating the correlation between RARS1 expression and vascular invasion. **(L)** Box plot showing the relationship between RARS1 expression and AFP levels. **(I)** TIDE analysis indicating that Cluster 1 patients have a poorer response to immune checkpoint blockade (ICB) therapy. Data were shown as mean ± SD. *p < 0.05, **p < 0.01, ***p < 0.001.

To further explore the prognostic significance of AR-DEGs in LIHC, we performed LASSO-Cox regression analysis on 10 AR-DEGs. Multivariate analysis identified IL18RAP, RARS1, CDKN2A, and CAPG as independent prognostic risk factors for LIHC (**Supplementary Figure 1A**). Given that IL18RAP, CDKN2A, and CAPG have been extensively studied in LIHC (17–19) and RARS1 shows a relatively high expression level in cluster 1(**Supplementary Figure 1**), we selected RARS1 as our research focus. Pan-cancer analysis of TCGA datasets revealed that RARS1 expression was significantly elevated in 17 cancer types, including LIHC (**Figure 2A**). Paired sample analysis confirmed that RARS1 was markedly upregulated in LIHC tumor tissues (**Figure 2B**). Further validation using multiple LIHC datasets consistently showed significant upregulation of RARS1 (**Figure 2C**). Single-cell RNA sequencing analysis demonstrated that RARS1 was predominantly expressed in malignant tumor cells (**Figure 2D**). Receiver operating characteristic (ROC) curve analysis indicated that RARS1 had strong diagnostic performance for LIHC (**Figure 2E**). Survival analysis revealed that high RARS1 expression was associated with poorer overall survival (OS) and disease-free survival (DFS) (**Figures 2F, G**). ROC curve analysis further confirmed that RARS1 effectively predicted 1-, 3-, and 5-year survival rates (**Figure 2H**). Clinical feature analysis indicated that RARS1 expression correlated with T stage, tumor grade, and radiotherapy status in LIHC patients (**Figures 2I–L**). After obtaining spatial transcriptomic data of LIHC from the GEO database, we first annotated the cells on the tissue slices of the reference group using a comprehensive single-cell RNA (scRNA) reference library. Following this, we analyzed the expression levels of RARS1 across different cell types within the tissue slices. The results revealed significantly higher expression of RARS1 in tumor cells compared to other cell types, indicating its prominent role in the tumor microenvironment (**Supplementary Figures 1B, C**).

### Validation of RARS1 expression in clinical and cell line samples

**Figure 3:**
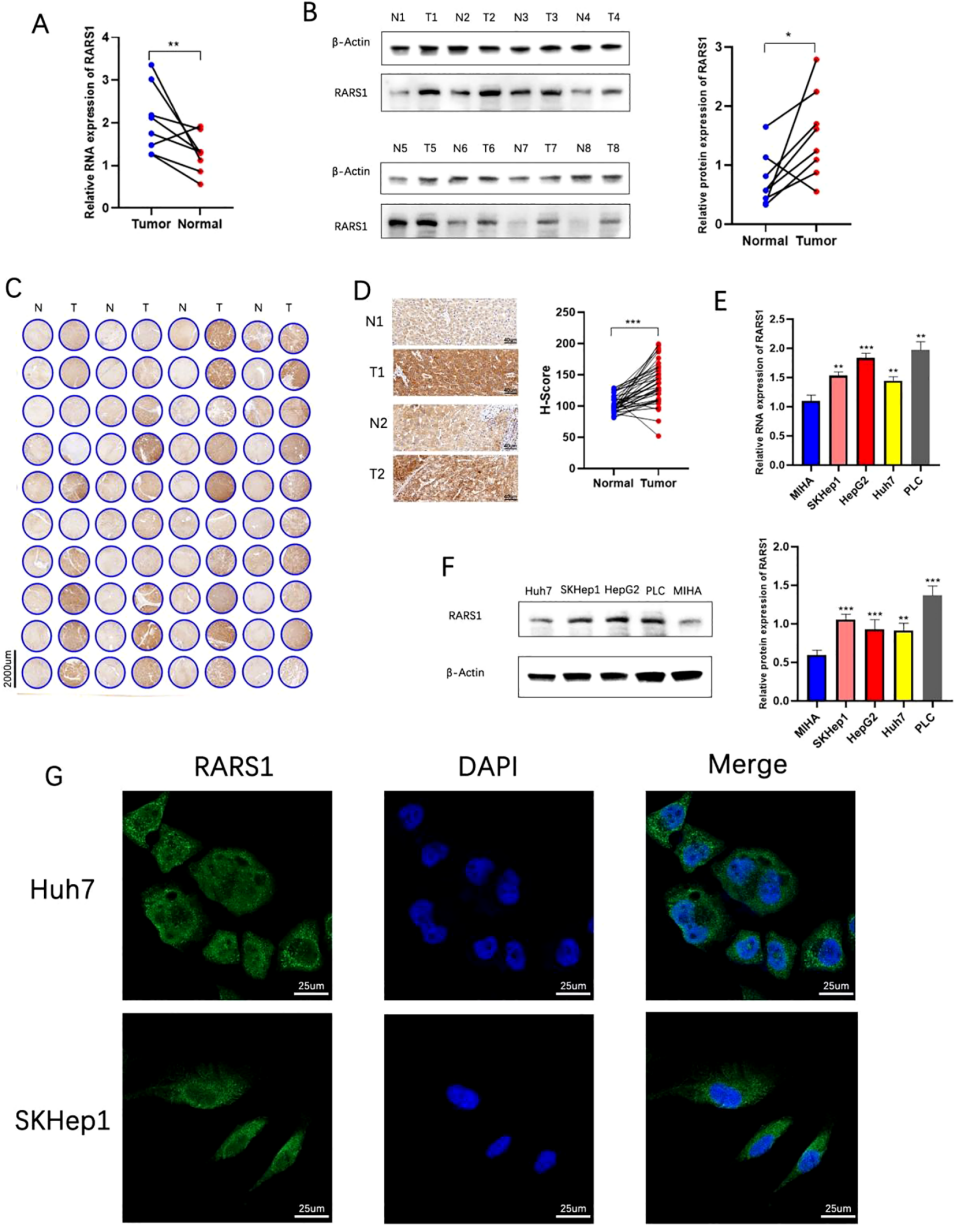
Validation of RARS1 expression in clinical and cell line samples. **(A, B)** RT-PCR and Western blot analyses demonstrated a significant upregulation of RARS1 mRNA **(A)** and protein **(B)** levels in liver cancer tissues (T) compared to adjacent normal tissues (N). **(C, D)** Tissue microarray analysis further confirmed the elevated expression of RARS1 in tumor tissues (T) compared to normal liver samples (N). Representative images **(C)** and quantification **(D)** of RARS1 immunohistochemical staining show a significant increase in RARS1 staining in tumors, with H-score values indicating a marked upregulation. **(E, F)** RARS1 expression was significantly higher in four LIHC cell lines (HepG2, PLC, SKHep1, and Huh7) compared to the normal hepatocyte cell line MIHA, as measured by RT-PCR **(E)** and Western blot **(F)**. **(G)** Immunofluorescence staining revealed that RARS1 is predominantly localized in the cytoplasm in both SKHep1 and Huh7 cells, with minimal nuclear expression. RT-PCR, Western blot, and immunohistochemistry analyses were performed using three independent biological replicates (n = 3). Data were shown as mean ± SD. *p < 0.05, **p < 0.01, ***p < 0.001.

Western blot and RT-PCR analyses demonstrated that RARS1 protein and mRNA levels were significantly upregulated in LIHC tumor tissues (**Figures 3A, B**). Tissue microarray staining further confirmed elevated RARS1 expression in tumor samples (**Figures 3C, D**). Expression analysis in hepatic cell lines revealed that RARS1 was significantly upregulated in four LIHC cell lines (HepG2, PLC, SKHep1, and Huh7) compared to normal hepatocyte MIHA cells (**Figures 3E, F**). Given that HepG2 originates from hepatoblastoma rather than hepatocellular carcinoma, we selected SKHep1 and Huh7 for further studies. Immunofluorescence staining indicated that RARS1 was predominantly localized in the cytoplasm, with minimal nuclear expression (**Figure 3G**).

### Knockdown of RARS1 inhibits LIHC cell growth, proliferation, and migration

**Figure 4:**
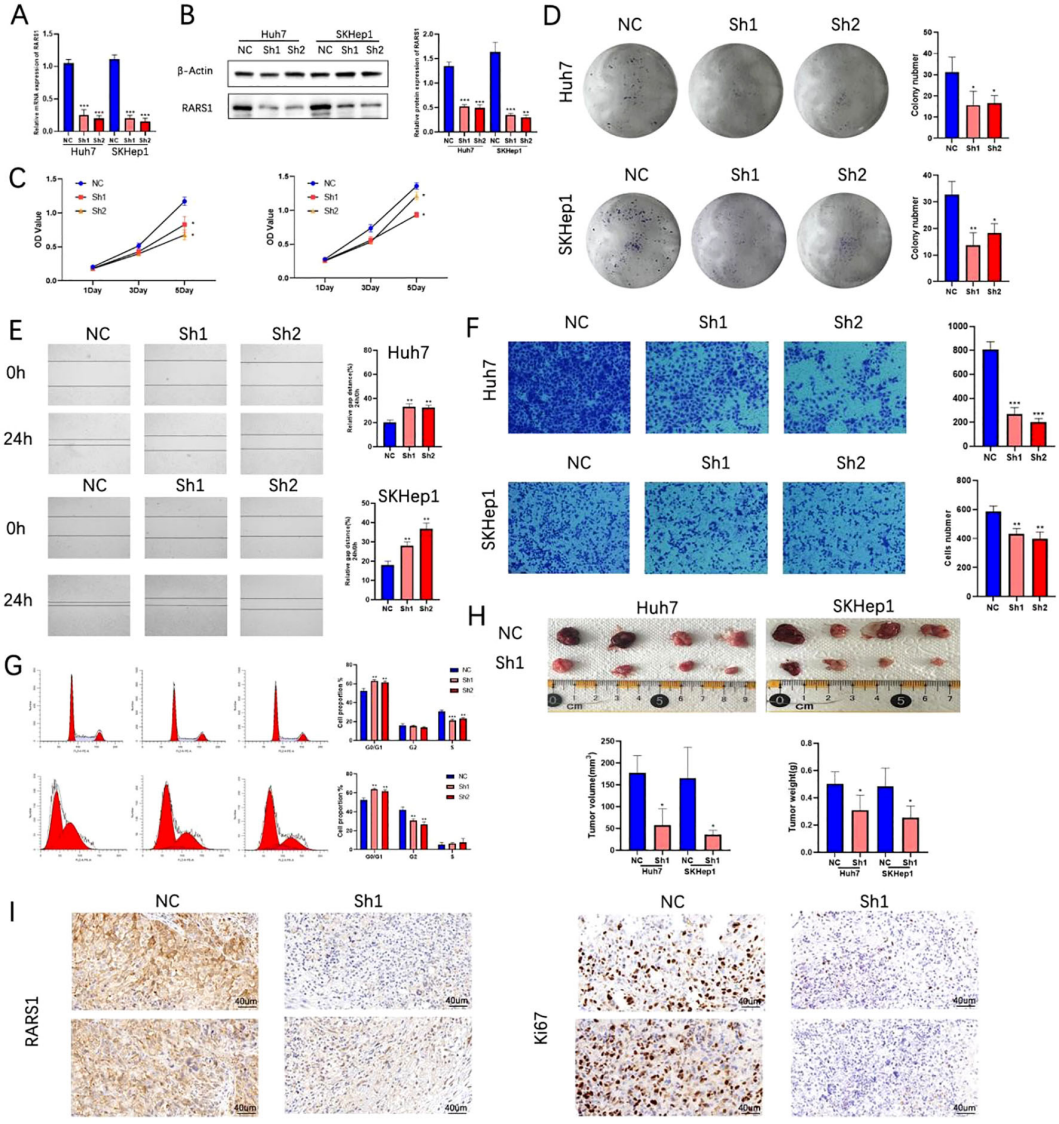
Knockdown of RARS1 inhibits LIHC cell growth, proliferation, and migration. **(B)** qRT-PCR **(A)** and Western blot **(B)** confirmed significant knockdown of RARS1 protein in Huh7 and SKHep1 cells after shRNA treatment. β-actin was used as a loading control. **(C)** Growth curve analysis showed reduced cell proliferation in RARS1 knockdown cells. **(D)** Colony formation assays revealed a significant decrease in colony numbers in knockdown cells. **(E)** Scratch wound healing assays showed delayed wound closure in RARS1 knockdown cells at 24 hours. **(F)** Transwell assays demonstrated significantly reduced cell invasion in knockdown cells. **(G)** Cell cycle analysis showed G0/G1 phase arrest in knockdown cells. **(H)** Subcutaneous tumor xenografts revealed smaller and lighter tumors in RARS1 knockdown groups. **(I)** Immunohistochemistry showed reduced Ki67 and RARS1 expression in knockdown tumors. Each experiment was independently repeated at least three times (n = 3). Data were shown as mean ± SD. *p < 0.05, **p < 0.01, ***p < 0.001.

Two RARS1-targeting shRNA lentiviruses were designed and transfected into SKHep1 and Huh7 cells. After 21 days of puromycin selection, stable knockdown cell lines were established. qRT-PCR and Western blot analyses confirmed significant reduction in RARS1 protein levels following shRNA treatment (**Figures 4A, B**). Growth curve and colony formation assays revealed that RARS1 knockdown significantly suppressed LIHC cell proliferation and colony-forming ability (**Figures 4C, D**). In scratch wound healing assays, knockdown cells exhibited slower wound closure (**Figure 4E**). Transwell assays further demonstrated that RARS1 knockdown significantly reduced invasive cell numbers (**Figure 4F**). Cell cycle analysis revealed that RARS1 suppression arrested tumor cells at the G0/G1 phase (**Figure 4G**). *In vivo* subcutaneous tumor xenograft models showed that tumors in the RARS1 knockdown group were significantly smaller and lighter than those in the control group (**Figure 4H**). Immunohistochemistry analysis indicated reduced Ki67 and RARS1 expression in the knockdown group (**Figure 4I**). These results suggest that RARS1 downregulation significantly inhibits LIHC cell growth, proliferation, and migration.

### RARS1 regulates the PI3K/p-AKT/GSK3β signaling pathway

**Figure 5:**
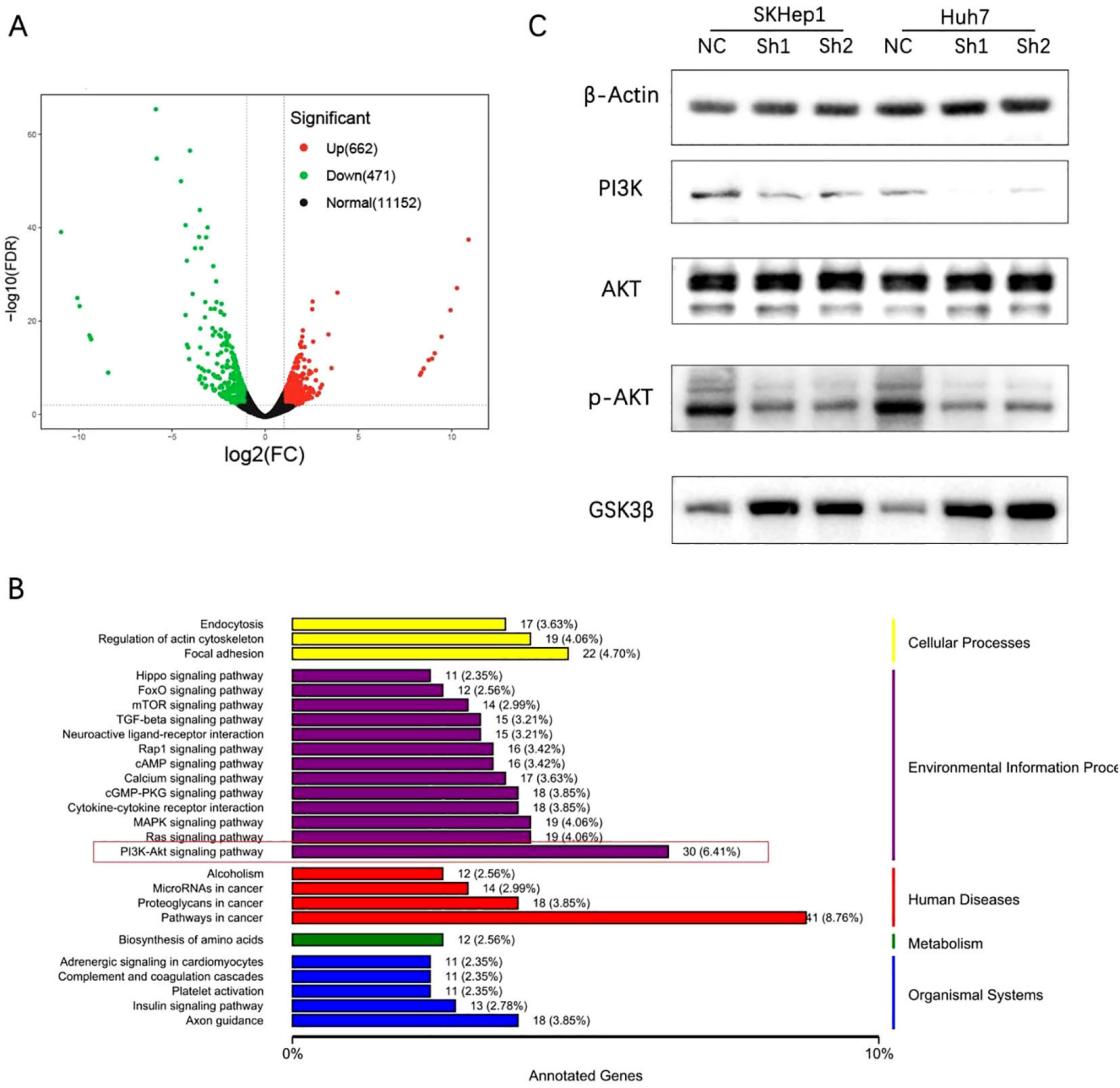
RARS1 regulates the PI3K/p-AKT/GSK3β signaling pathway. **(A)** RNA-seq analysis identified 662 upregulated and 471 downregulated genes in RARS1-knockdown SKHep1 cells, shown in a volcano plot. **(B)** KEGG pathway analysis revealed enrichment in focal adhesion, PI3K-AKT signaling, and cancer pathways. **(C)** Western blot analysis showed that RARS1 knockdown reduced PI3K and p-AKT expression while increasing GSK3β levels in SKHep1 and Huh7 cells. Western blot analyses were performed in triplicate (n = 3).

Considering the high expression level of RARS1 in SKHep1 cells and their strong metastatic potential, we selected SKHep1 for RNA sequencing. RNA-seq analysis was performed on control and RARS1-knockdown cells. RNA-seq analysis was performed on SKHep1 control and RARS1-knockdown cells. DEGs were identified using |log2FC| > 1 and *p* < 0.05, revealing 662 upregulated and 471 downregulated genes (**Figure 5A**). KEGG enrichment analysis indicated that RARS1 was closely associated with Focal adhesion, PI3K-AKT signaling, and pathways in cancer (**Figure 5B**). Western blot analysis showed that RARS1 knockdown reduced PI3K and phosphorylat-AKT(p-AKT) levels, while increasing GSK3β expression, suggesting that RARS1 modulates the PI3K/p-AKT/GSK3β signaling pathway in tumor proliferation and migration (**Figure 5C**).

### RARS1 interacts with ENO1 and regulates its ubiquitination

**Figure 6:**
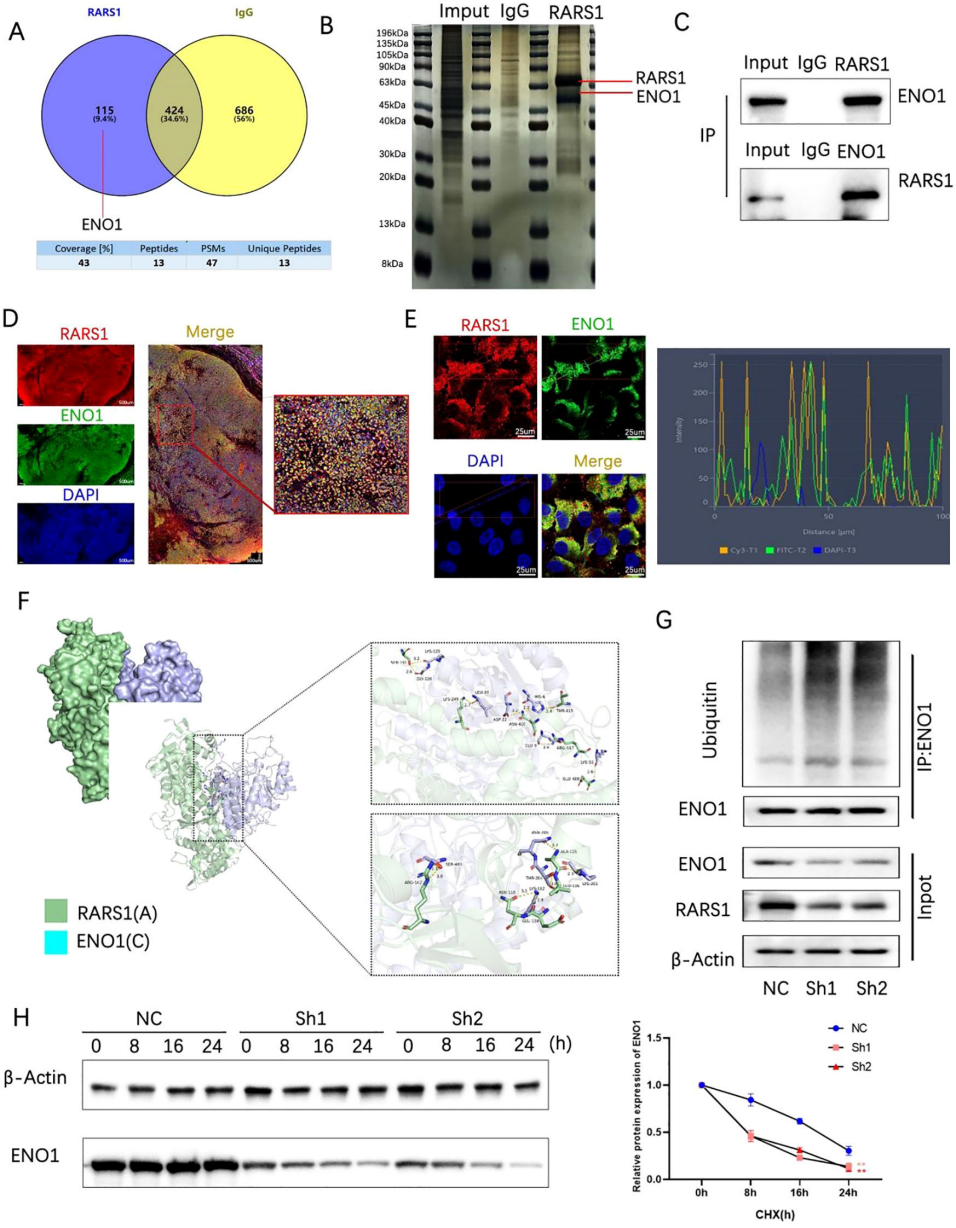
RARS1 interacts with ENO1 and regulates its ubiquitination. **(A)** Venn diagram showing the overlap of proteins interacting with RARS1, identified through mass spectrometry. A total of 115 proteins were found to interact with RARS1, with 424 proteins identified through IgG control and 686 proteins through ENO1 interaction. **(B)** Silver-stained SDS-PAGE showing co-immunoprecipitation of RARS1 and ENO1. The bands corresponding to RARS1 and ENO1 are indicated. **(C)** Co-immunoprecipitation of RARS1 and ENO1 in LIHC cells. The inputs, IgG controls, and immunoprecipitates are shown, confirming their interaction. **(D)** Immunofluorescence staining of LIHC tissue sections showing colocalization of RARS1 (red) and ENO1 (green). DAPI (blue) marks the nuclei. High magnification reveals their proximity in the cytoplasm. **(E)** Immunofluorescence analysis of RARS1 and ENO1 in LIHC cells, with colocalization shown in merged images. The intensity profile (right) demonstrates their spatial overlap, indicating close interaction in the cells. **(F)** Molecular docking analysis of the binding interface between RARS1 (green) and ENO1 (cyan). The detailed view of the binding sites and critical residues in the interaction is shown. **(G)** Ubiquitination assays demonstrating that RARS1 knockdown (Sh1, Sh2) leads to increased ENO1 ubiquitination. The blot shows enhanced ubiquitin-conjugated ENO1 protein levels in the knockdown samples. **(H)** Cycloheximide (CHX) chase assay showing that RARS1 knockdown significantly reduces ENO1 protein stability. The protein degradation curve (right) illustrates a faster degradation rate of ENO1 in Sh1 and Sh2 cells compared to NC control. All co-immunoprecipitation, immunofluorescence, and ubiquitination assays were independently repeated three times (n = 3). Data were shown as mean ± SD. **p < 0.01.

Immunoprecipitation and mass spectrometry identified 115 proteins interacting with RARS1 (**Figure 6A**). Given that ENO1 is highly expressed in various tumors and that mass spectrometry analysis has achieved high peptide counts and coverage rates, we selected ENO1 as a potential interacting protein of RARS1. Silver staining and co-immunoprecipitation confirmed significant interaction between RARS1 and ENO1 (**Figures 6B, C**). Immunofluorescence staining demonstrated colocalization of RARS1 and ENO1 in LIHC tissues and cells (**Figures 6D, E**). Molecular docking analysis revealed strong binding sites between ENO1 and RARS1 (**Figure 6F**). Ubiquitination assays indicated that RARS1 knockdown significantly increased ENO1 degradation (**Figure 6G**). Cycloheximide (CHX) chase assays further confirmed that RARS1 knockdown reduced ENO1 protein stability (**Figure 6H**), highlighting its crucial role in ENO1 regulation.

### RARS1 suppresses ferroptosis via ENO1 in LIHC cells

**Figure 7:**
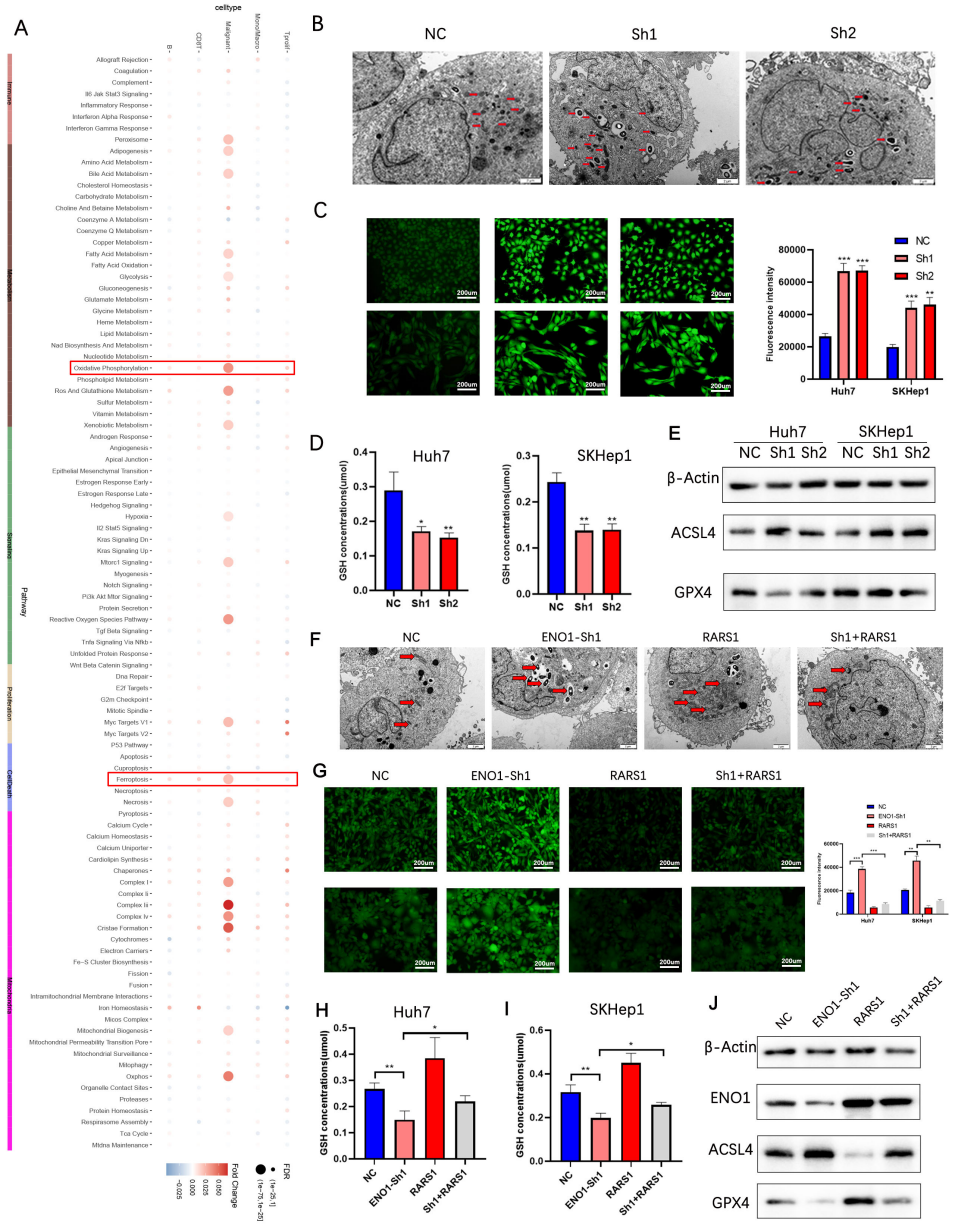
RARS1 suppresses ferroptosis via ENO1 in LIHC cells. **(A)** Single-cell AUCell scoring analysis showing enrichment of oxidative phosphorylation and ferroptosis pathways in RARS1-positive malignant cells. The heatmap indicates the relative expression of key ferroptosis-related genes. **(B)** Transmission electron microscopy (TEM) images showing mitochondrial morphological changes upon RARS1 knockdown (Sh1, Sh2). Mitochondrial shrinkage and increased membrane density are observed in the knockdown groups (red arrows). **(C)** ROS assay in Huh7 and SKHep1 cells demonstrating significantly increased ROS levels upon RARS1 knockdown (Sh1, Sh2) compared to the NC group. **(D)** GSH assay showing significantly reduced GSH levels in RARS1 knockdown cells (Sh1, Sh2) in both Huh7 and SKHep1 cells. **(E)** Western blot analysis revealing decreased GPX4 and increased ACSL4 expression in RARS1 knockdown cells, confirming the induction of ferroptosis. **(F)** TEM images showing mitochondrial changes after ENO1 knockdown (ENO1-Sh1) and RARS1 overexpression (RARS1) in Huh7 and SKHep1 cells. Mitochondrial morphological alterations are indicated by red arrows, and RARS1 overexpression partially reverses the effects of ENO1 knockdown. **(G)** ROS assay showing enhanced ROS levels in ENO1 knockdown (ENO1-Sh1) cells, which were partially reversed by RARS1 overexpression. Quantification of ROS intensity is shown in the right panel. **(H)** GSH assay in Huh7 cells confirming the reduction of GSH levels in ENO1-knockdown cells (ENO1-Sh1), which was partially reversed by RARS1 overexpression (RARS1). **(I)** GSH assay in SKHep1 cells showing similar effects as in Huh7, where RARS1 overexpression mitigated the decrease in GSH levels caused by ENO1 knockdown. **(J)** Western blot analysis in Huh7 and SKHep1 cells showing the expression of ENO1, ACSL4, and GPX4 after RARS1 overexpression and ENO1 knockdown. RARS1 overexpression restores ENO1 expression and reduces ACSL4 while increasing GPX4 expression. All experiments (TEM, ROS, GSH, and Western blot) were performed in triplicate (n = 3). Data were shown as mean ± SD. *p < 0.05, **p < 0.01, ***p < 0.001.

Single-cell AUCell scoring suggested that oxidative phosphorylation and ferroptosis pathways were enriched in RARS1-positive malignant cells (**Figure 7A**). Transmission electron microscopy revealed that RARS1 knockdown induced mitochondrial shrinkage and increased membrane density (**Figure 7B**). ROS and GSH assays confirmed increased ROS and decreased GSH levels upon RARS1 knockdown (**Figures 7C, D**). Western blot analysis indicated reduced GPX4 and increased ACSL4 expression, confirming ferroptosis induction (**Figure 7E**). ENO1 knockdown further enhanced ferroptosis, while RARS1 overexpression partially reversed these effects (**Figures 7F–J**).

### RARS1 knockdown inhibits macrophage M2 polarization in LIHC tumor microenvironment

**Figure 8:**
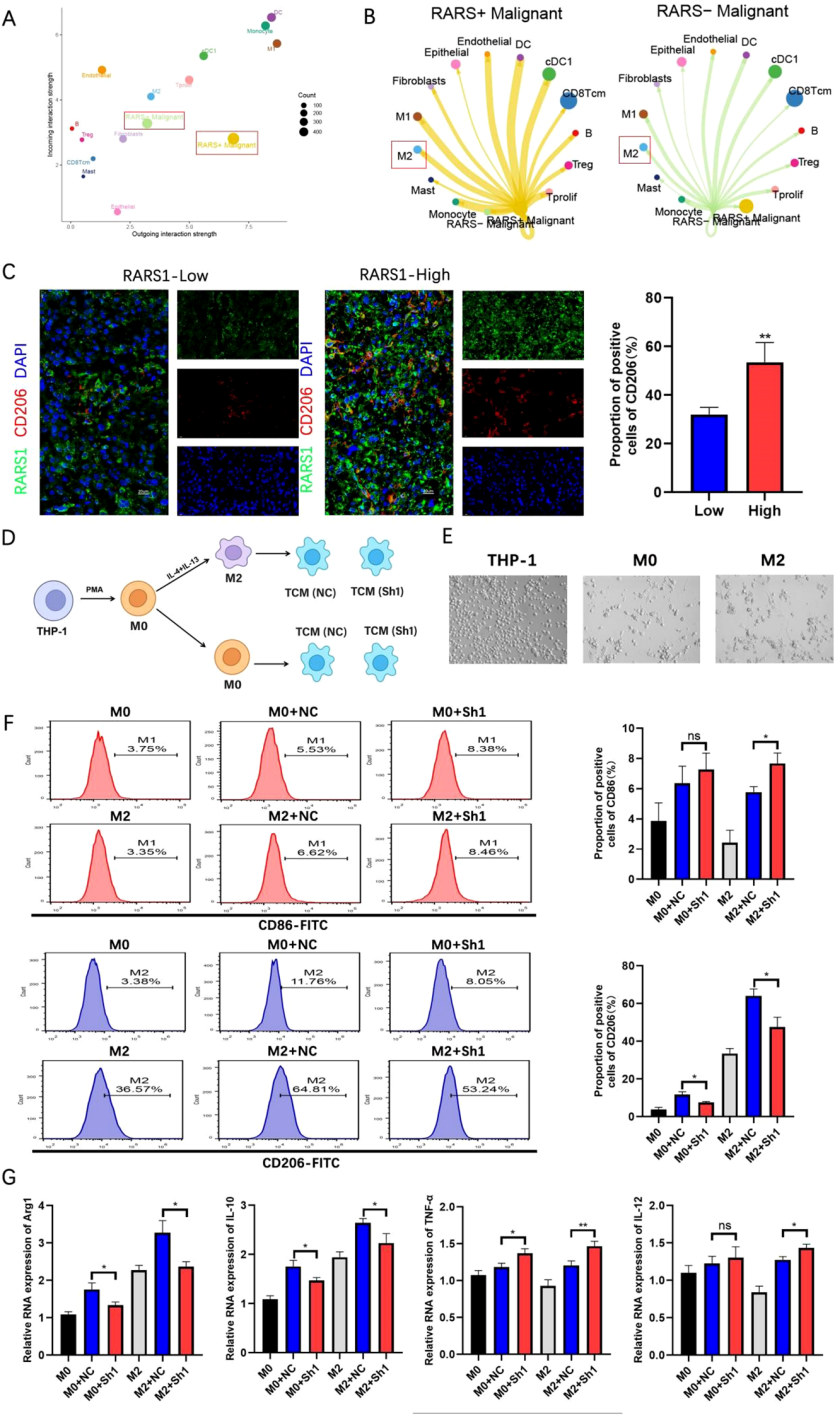
RARS1 knockdown inhibits macrophage M2 polarization in the LIHC tumor microenvironment. **(A)** Single-cell communication analysis of RARS1-positive and RARS1-negative malignant tumor cells based on TCGA LIHC cohort. The plot shows the relative communication strength and frequency between tumor cells and other cell types, with RARS1-positive cells demonstrating stronger and more frequent signal secretion, particularly with macrophages. **(B)** Cell-cell communication analysis revealing stronger interaction between RARS1-positive cells and M2 macrophages, suggesting RARS1 enhances macrophage differentiation and communication in the tumor microenvironment. **(C)** Multiplex immunofluorescence staining of HCC tissues showing higher CD206^+^ macrophage infiltration in RARS1-high tumors than in RARS1-low tumors (P < 0.01, Student’s t-test). **(D)** Experimental workflow: THP-1 cells were induced with PMA to M0, further polarized to M2 by IL-4/IL-13, and then cultured with tumor-conditioned media (TCM) from control (NC) or RARS1-knockdown (Sh1) SKHep1 cells. **(E)** Representative morphology of THP-1, M0, and M2 macrophages under phase-contrast microscopy. **(F)** Flow cytometry analysis showing that RARS1-knockdown TCM increased CD86^+^(M1) and decreased CD206^+^(M2) macrophages compared with NC-TCM. **(G)** qRT-PCR showing downregulation of M2 genes (Arg1, IL10) and upregulation of M1 genes (TNFα, IL12A) in RARS1-knockdown TCM-treated macrophages. All flow cytometry, qRT-PCR, and ELISA assays were performed with three independent biological replicates (n = 3). Data were shown as mean ± SD. *p < 0.05, **p < 0.01. ns: No statistical significance.

To explore the role of RARS1 in the LIHC tumor microenvironment, we analyzed immune infiltration in the TCGA LIHC cohort using the ssGSEA algorithm provided by the GSVA package. The results showed a positive correlation between RARS1 expression and Th2 cells, T helper cells, and macrophages (**Supplementary Figure 2A**). Spatial transcriptomics analysis revealed a significant correlation between RARS1 and macrophages as well as fibroblasts (**Supplementary Figure 2B**). These findings suggest that RARS1 may participate in the modulation of the LIHC tumor microenvironment by affecting macrophage differentiation. Based on single-cell data, we classified malignant tumor cells into RARS1-positive and RARS1-negative groups and analyzed their communication strength and frequency with other cells. The cell communication analysis revealed that RARS1-positive cells exhibited stronger and more frequent signal secretion, while RARS1-negative cells demonstrated enhanced signal reception (**Figure 8A**), particularly with M2 macrophages (**Figure 8B**), suggesting that RARS1 enhances macrophage interactions. Furthermore, RARS1-positive cells likely secrete stronger signaling molecules, influencing the tumor immune microenvironment (**Supplementary Figure 2C**). Consistent with these computational predictions, multiplex immunofluorescence staining of HCC tissues demonstrated that RARS1-high tumors displayed a markedly higher proportion of CD206^+^ macrophages compared with RARS1-low tumors (**Figure 8C**). This observation indicates that RARS1 upregulation correlates with enhanced infiltration of M2-polarized macrophages, supporting a pro-tumorigenic immune microenvironmen (20, 21). To experimentally model this phenomenon, we established a THP-1 polarization system using tumor-conditioned media (TCM) derived from either control (NC) or RARS1-knockdown (Sh1) SKHep1 cells (**Figure 8D**). Morphological assessment under phase-contrast microscopy (**Figure 8E**) showed that THP-1 cells transitioned from a round monocyte-like morphology (THP-1) to adherent M0 and spindle-shaped M2 macrophages following PMA and IL-4/IL-13 induction, respectively. Flow cytometry analysis further confirmed that RARS1 depletion in SKHep1-derived TCM profoundly influenced macrophage polarization. Specifically, TCM from RARS1-knockdown cells significantly reduced the population of CD206^+^(M2) macrophages (22), while increasing CD86^+^(M1) macrophages (23), compared with TCM from control cells (**Figure 8F**). qRT-PCR analysis of macrophage-associated genes revealed that RARS1-knockdown TCM significantly downregulated M2-associated transcripts (Arg1 and IL10) (24), while upregulating M1-related genes (TNFα and IL12A) (25) (**Figure 8G**). Together, these data demonstrate that RARS1-expressing tumor cells orchestrate macrophage polarization toward an immunosuppressive, M2-like state, whereas the loss of RARS1 disrupts this crosstalk and promotes a shift toward a proinflammatory, antitumor macrophage phenotype.

### AH-6809 inhibits RARS1 expression and suppresses LIHC cell proliferation, migration, and invasion

**Figure 9:**
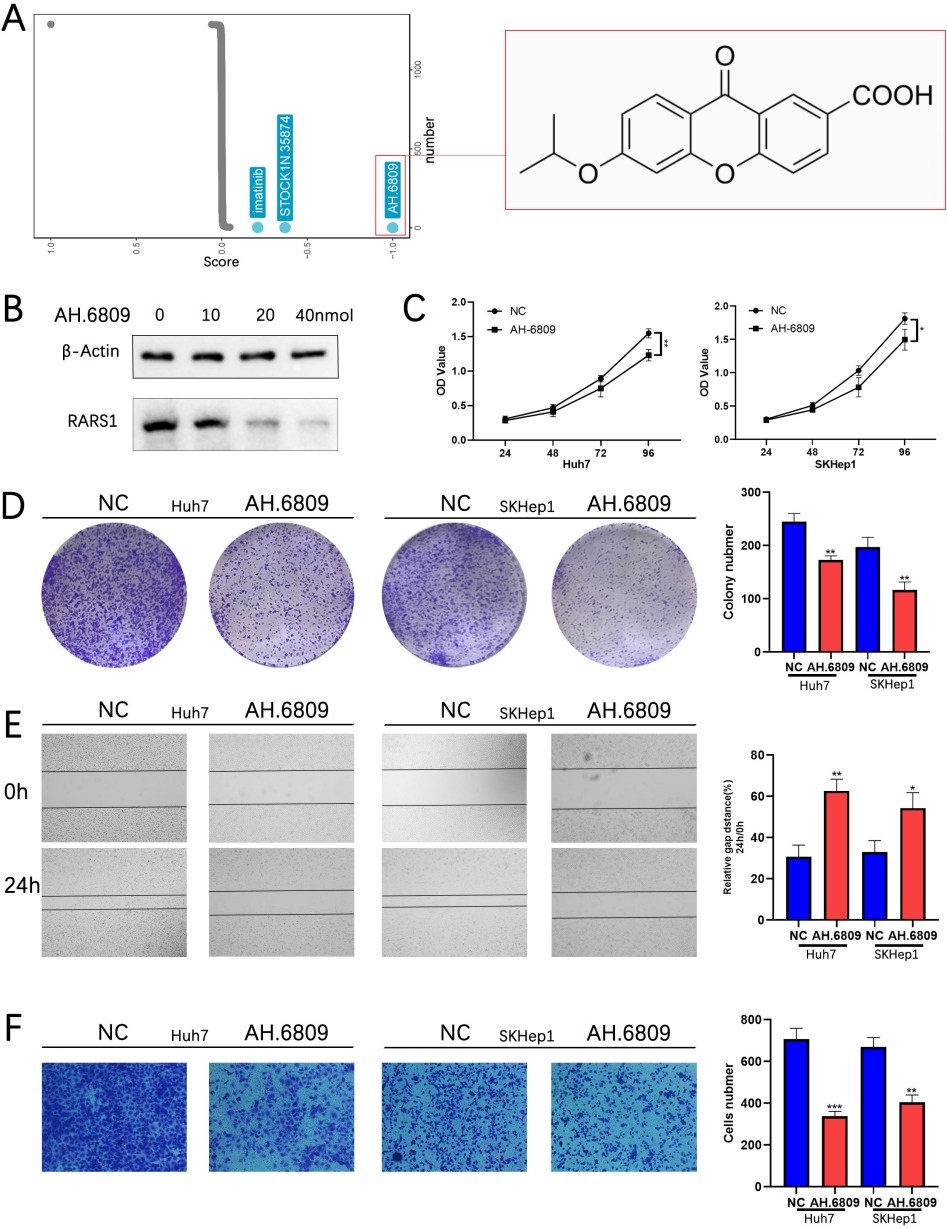
AH-6809 inhibits RARS1 expression and suppresses LIHC cell proliferation, migration, and invasion. **(A)** Connectivity Map (cMAP) analysis identifying potential small-molecule inhibitors targeting RARS1. AH-6809 showed a strong negative correlation with the RARS1 gene expression signature and was selected for further validation. **(B)** Western blot analysis of RARS1 protein levels in Huh7 cells treated with increasing concentrations of AH-6809 (0, 10, 20, and 40 nmol). RARS1 expression decreased in a dose-dependent manner. **(C)** Cell proliferation assessed by CCK-8 assay in Huh7 and SKHep1 cells treated with 20 nmol AH-6809 for 24–96 (h) AH-6809 significantly reduced cell viability compared with the control group. **(D)** Colony formation assay showing that AH-6809 markedly decreased the number and size of colonies in Huh7 and SKHep1 cells. **(E)** Wound-healing assay showing inhibition of cell migration after 24 h of AH-6809 treatment. **(F)** Transwell invasion assay showing that AH-6809 significantly reduced the number of invasive cells in both Huh7 and SKHep1 lines. All cell-based assays (CCK-8, colony formation, wound-healing, Transwell, and Western blot) were conducted in triplicate (n = 3). Data were shown as mean ± SD. *p < 0.05, **p < 0.01, ***p < 0.001.

Subsequently, drug sensitivity analysis using the GSCA database revealed that RARS1 is closely associated with resistance to several chemotherapy agents (**Supplementary Figure 3A**). To identify potential small-molecule inhibitors targeting RARS1, we performed a Connectivity Map (cMAP) analysis, which revealed AH-6809 as one of the top compounds negatively correlated with the RARS1 expression signature (**Figure 9A**). Molecular docking analysis further revealed multiple binding sites between AH.6809 and RARS1 (**Supplementary Figure 3B**). Western blot analysis further demonstrated that RARS1 protein levels gradually decreased with increasing concentrations of AH-6809 (0–40 nmol) in Huh7 cells (**Figure 9B**), suggesting a dose-dependent inhibition of RARS1 expression. To evaluate the biological effects of AH-6809 on hepatocellular carcinoma (LIHC) cells, we treated Huh7 and SKHep1 cells with 20 nmol AH-6809 and assessed cell viability. CCK-8 assays revealed that AH-6809 significantly reduced cell proliferation in both cell lines in a time-dependent manner compared with control groups (**Figure 9C**). Consistent with this, colony formation assays showed a marked decrease in the number and size of colonies following AH-6809 treatment (**Figure 9D**), indicating a long-term suppressive effect on clonogenic capacity. Similarly, wound-healing assays demonstrated that AH-6809 significantly impaired cell migration after 24 hours of treatment (**Figure 9E**). In addition, Transwell invasion assays revealed a substantial reduction in the number of invasive cells upon AH-6809 exposure (**Figure 9F**). Together, these results indicate that AH-6809 acts as a potential inhibitor of RARS1, effectively suppressing hepatocellular carcinoma cell proliferation, migration, and invasion, thereby highlighting its therapeutic potential in targeting RARS1-driven tumor progression.

## Discussion

Liver hepatocellular carcinoma (LIHC) remains one of the most aggressive and common forms of cancer, with high morbidity and mortality rates worldwide. Despite advances in early diagnosis and treatment strategies, the therapeutic challenge persists due to high recurrence rates and resistance to conventional therapies, underscoring the need for novel biomarkers and therapeutic targets (26).

The relationship between allograft rejection-related genes (ARRGs) and cancer progression is a novel area of investigation. In this study, we identified key AR-DEGs, including RARS1, which not only play critical roles in transplant rejection but also appear to contribute to the tumorigenic process in LIHC. By performing NMF clustering on AR-DEGs, we classified LIHC patients into distinct molecular subtypes, which showed significant differences in prognosis and immune infiltration. The molecular subtypes identified through AR-DEGs provide valuable insights into the complex interplay between immune rejection mechanisms and tumor progression in LIHC. Notably, patients in Cluster 1, characterized by higher immune infiltration and stemness properties, exhibited a worse prognosis and poor response to immune checkpoint blockade (ICB) therapy, as determined by TIDE analysis. These findings suggest that ARRGs, including RARS1, can help classify LIHC patients into subgroups with distinct immune profiles and therapeutic responses. This molecular subclassification may pave the way for personalized treatment strategies, particularly in the context of immunotherapy, by identifying patients who may benefit from immune modulation or targeted therapies (27).

RARS1 expression is significantly upregulated in LIHC tissues, as demonstrated by multiple independent datasets, including TCGA and patient-derived clinical samples. High RARS1 expression correlates with poor overall survival (OS) and disease-free survival (DFS), suggesting that RARS1 may serve as a prognostic marker for LIHC. Pan-cancer analysis further highlighted that RARS1 is upregulated in various cancer types, reinforcing its potential as a universal oncogene. Single-cell RNA sequencing analysis revealed that RARS1 is predominantly expressed in malignant tumor cells, with its expression being significantly higher in these cells compared to normal liver cells. This suggests that RARS1 may play an essential role in maintaining the malignant phenotype of LIHC, further supporting its potential as a therapeutic target. Moreover, high RARS1 expression was found to be associated with unfavorable clinical features, such as advanced TNM stage and poor tumor differentiation.

Functional experiments demonstrated that RARS1 knockdown significantly inhibited the growth, proliferation, and migration of LIHC cells. Both *in vitro* assays, including colony formation and scratch wound healing, as well as *in vivo* tumor xenograft models, showed a reduction in tumor growth and volume upon RARS1 suppression. The PI3K/AKT/GSK3β signaling pathway is pivotal in regulating various aspects of tumor biology, including cell growth, metabolism, and migration (28, 29). In our study, RNA-seq analysis identified significant alterations in this pathway following RARS1 knockdown, with decreased PI3K and p-AKT levels and increased GSK3β expression. The PI3K/AKT pathway is often dysregulated in cancer, contributing to uncontrolled tumor cell growth and survival (30). The downregulation of AKT phosphorylation and upregulation of GSK3β in RARS1-deficient cells suggests a shift toward a less proliferative and more apoptosis-prone phenotype. This finding provides novel mechanistic insights into how RARS1 might regulate tumorigenesis and highlights the potential of targeting the PI3K/AKT pathway for therapeutic intervention in LIHC.

Ferroptosis, a form of regulated cell death driven by iron accumulation and lipid peroxidation, has emerged as an important mechanism in cancer progression and therapy resistance (31, 32). Our study provides evidence that RARS1 modulates ferroptosis in LIHC cells, as RARS1 knockdown increased ROS levels and decreased GSH levels, leading to mitochondrial damage, which are hallmarks of ferroptosis (33, 34). Additionally, we observed that ENO1, an enzyme involved in cellular metabolism, interacts with RARS1 and plays a critical role in regulating ferroptosis. Specifically, RARS1 seems to inhibit ferroptosis through ENO1, as ENO1 knockdown further enhanced ferroptotic cell death in RARS1-deficient cells. This suggests that RARS1 may protect LIHC cells from ferroptosis through its regulation of ENO1 activity, thereby contributing to tumor cell survival. The role of ENO1 in cancer has been well-established, with studies showing that its overexpression promotes tumor progression, angiogenesis, and resistance to ferroptosis (35, 36). Our findings provide further evidence that ENO1 may be a key mediator of RARS1’s protective effect against ferroptosis in LIHC cells. Targeting the RARS1-ENO1 axis could offer a novel therapeutic strategy for inducing ferroptosis in LIHC, particularly in tumor cells resistant to conventional therapies.

Another striking finding of our study is the role of RARS1 in modulating the immune microenvironment of LIHC. We observed that RARS1 expression correlates with the infiltration of various immune cells, particularly macrophages, in both TCGA cohorts and patient samples. RARS1-positive cells exhibited enhanced communication with immune cells, especially M2 macrophages, which are known to promote tumor progression and immune evasion (37, 38). Single-cell RNA sequencing and spatial transcriptomics further revealed that RARS1 positively regulates macrophage polarization toward the M2 phenotype, which is associated with immune suppression and cancer progression.

Our co-culture experiments confirmed that RARS1 knockdown shifted macrophage polarization from the M2 to the M1 phenotype, characterized by increased expression of pro-inflammatory cytokines such as TNF-α and IL-12 (25). These results suggest that RARS1 plays a crucial role in shaping the immune landscape of the LIHC tumor microenvironment by promoting immunosuppressive macrophage polarization. Previous studies have shown that targeting macrophage polarization can reverse immune suppression in the tumor microenvironment and enhance anti-tumor immunity (39, 40). Thus, RARS1 may represent a valuable target for modulating the immune response in LIHC and improving the efficacy of immunotherapies.

AH.6809 has demonstrated potent anti-tumor activity in various cancers (41–43). However, its effects in LIHC have not been previously reported. Using the GSCA database and cMAP analysis, we identified AH.6809 as a promising therapeutic agent capable of counteracting the tumor-promoting effects of RARS1. Treatment of Huh7 cells with AH.6809 led to a dose-dependent reduction in RARS1 protein levels, suggesting that AH.6809 may inhibit RARS1-mediated oncogenic activity *in vitro*. This finding provides a novel approach for developing targeted therapies aimed at suppressing the role of RARS1 in tumor progression.

While our study provides important insights into the role of RARS1 in LIHC, several limitations should be considered. First, although our findings are supported by multiple datasets and *in vitro*/*in vivo* experiments, further validation in larger, independent cohorts is necessary to confirm the clinical relevance of RARS1 as a prognostic marker. Second, while we demonstrate that RARS1 regulates immune cell polarization and ferroptosis, the exact molecular mechanisms through which RARS1 interacts with these processes remain unclear and warrant further investigation. Only female BALB/c nude mice were used in this study due to housing and welfare constraints. However, since sex differences may influence tumor progression and therapeutic response, this represents a limitation of our study. Future work will include both male and female cohorts to evaluate potential sex-specific effects on RARS1-mediated tumor biology.

## Conclusion

In conclusion, our study highlights the critical role of RARS1 in LIHC progression through its regulation of key oncogenic pathways, immune modulation, and ferroptosis. RARS1 not only serves as a prognostic biomarker for LIHC but also represents a promising therapeutic target for modulating tumor growth and improving immune responses. The integration of AR-DEGs to classify LIHC patients into molecular subtypes offers a new perspective on how immune rejection pathways intersect with cancer biology, potentially guiding the development of more effective, personalized therapies. Future studies are needed to explore the clinical application of RARS1-targeted therapies, either alone or in combination with immunotherapy and ferroptosis-inducing agents, to improve the outcomes for LIHC patients.

## Data Availability

The datasets generated for this study can be found in the https://www.ncbi.nlm.nih.gov/geo/. The names of the repository/repositories and accession number(s) can be found in the article/**Supplementary Material**.

## Supplementary material

Supplementary Figure 1 Spatial expression of RARS1 in the tumor microenvironment. **(A)** RARS1 expression levels across Clusters 1–3, showing significantly higher expression in Cluster 1. **(B)** Spatial transcriptomic maps showing the distribution of immune and stromal cells. RARS1 expression is predominantly enriched in tumor cells (outlined in red). **(C)** Bar graph showing mean RARS1 expression in malignant, mixed, and normal regions. Data were shown as mean ± SD. *: *p* < 0.05, **: *p* < 0.01, ***: *p* < 0.001.

Supplementary Figure 2 Immune infiltration and signaling patterns in the LIHC tumor microenvironment associated with RARS1. **(A)** Immune cell infiltration analysis in the TCGA LIHC cohort using the ssGSEA algorithm reveals a significant positive correlation between RARS1 expression and the abundance of Th2 cells, T helper cells, and macrophages. **(B)** Spatial transcriptomics analysis further confirmed a strong correlation between RARS1 and the presence of macrophages and fibroblasts within the tumor microenvironment. **(C)** Signaling pathway analysis based on the relative strength of outgoing and incoming signaling patterns in RARS1-positive cells. The heatmaps show the relative signaling strength for various pathways, with red shades representing strong outgoing signaling and green shades representing strong incoming signaling. Data were shown as mean ± SD. *: *p* < 0.05, **: *p* < 0.01, ***: *p* < 0.001.

Supplementary Figure 3 Drug sensitivity analysis and potential small molecule targeting RARS1. **(A)** Drug sensitivity analysis conducted using the GSCA database revealed a strong association between RARS1 expression and resistance to various chemotherapy agents. The correlation between RARS1 and drug sensitivity is visualized through a correlation matrix showing the relationship between gene expression and chemotherapy efficacy. **(B)** Molecular docking analysis of AH.6809 binding to RARS1 revealed multiple binding sites, including hydrogen bonding and hydrophobic interactions. The 3D structure of RARS1 is shown with AH.6809 binding in the active site, and the detailed molecular interactions between AH.6809 and RARS1 are illustrated.

Supplementary Table 1 Sequences of the QRT-PCR primers used for each gene.

Supplementary Table 2 Univariate and multivariate Cox proportional hazards regression analyses of the prognostic value of10 AR-DEGs in the TCGA LIHC cohort.
