# Correction: RARS1 inhibits ENO1 ubiquitination and degradation to protect against ferroptosis in hepatocellular carcinoma

**DOI:** 10.3389/fimmu.2026.1820769

**Published:** 2026-03-13

**Authors:** Shouge Zang, Jvlong Ma, Lichang Chen, Di Cui, Jiangtao Yu

**Affiliations:** 1 Department of Hepatopancreatobiliary Surgery, Fuyang People’s Hospital of Anhui Medical University, Fuyang, Anhui, China; 2 Clinical Laboratory, No.2 People’s Hospital of Fuyang City, Fuyang, Anhui, China; 3 Fuyang Medical College, Fuyang Normal University, Fuyang, Anhui, China

**Keywords:** arginyl-tRNA synthetase, ENO1, allograft rejection, ubiquitination, ferroptosis

There was a mistake in **Figure 9B** as published: the β-Actin band was incorrectly used during figure preparation. The corrected **Figure 9B** appears below:

**Figure 9:**
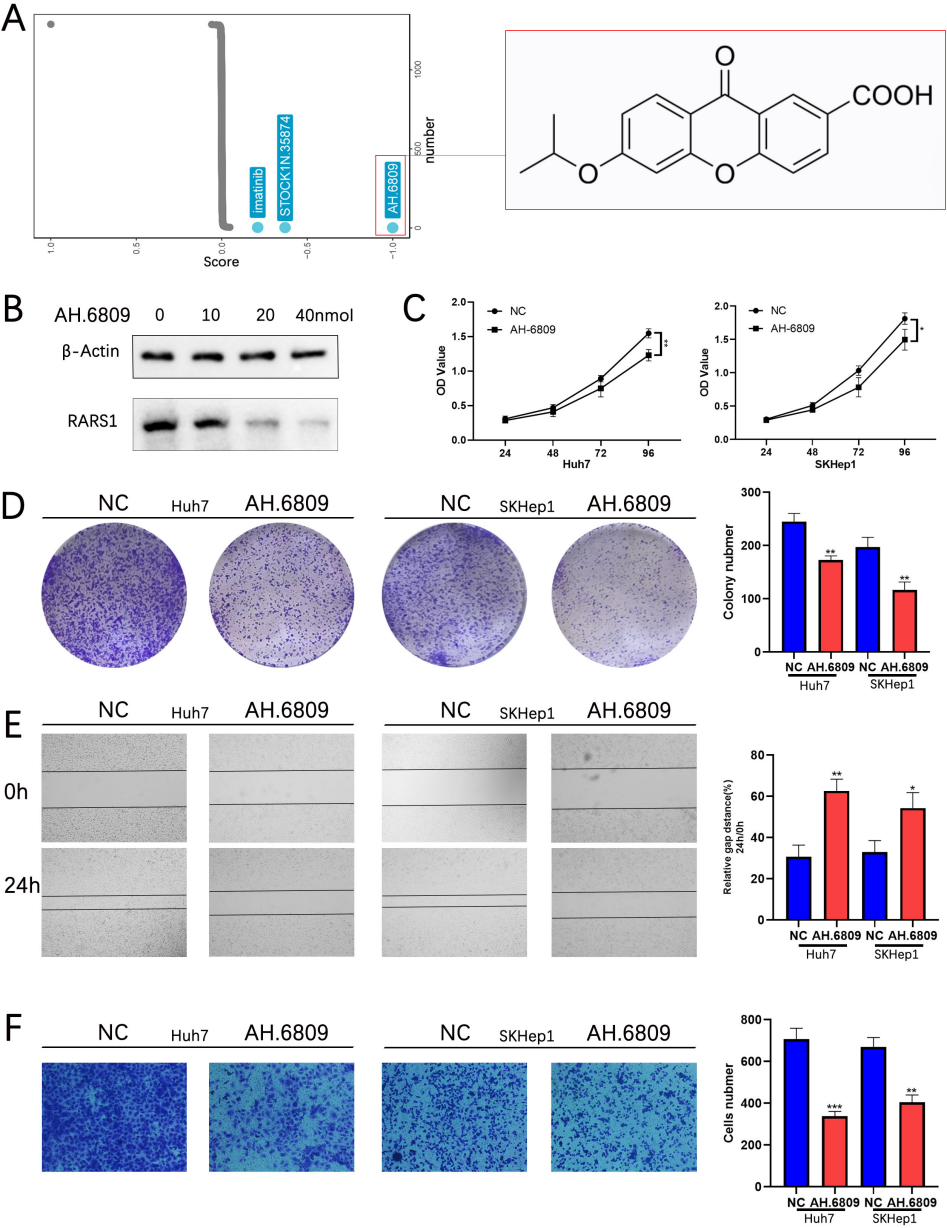
AH-6809 inhibits RARS1 expression and suppresses LIHC cell proliferation, migration, and invasion. **(A)** Connectivity Map (cMAP) analysis identifying potential small-molecule inhibitors targeting RARS1. AH-6809 showed a strong negative correlation with the RARS1 gene expression signature and was selected for further validation. **(B)** Western blot analysis of RARS1 protein levels in Huh7 cells treated with increasing concentrations of AH-6809 (0, 10, 20, and 40 nmol). RARS1 expression decreased in a dose-dependent manner. **(C)** Cell proliferation assessed by CCK-8 assay in Huh7 and SKHep1 cells treated with 20 nmol AH-6809 for 24–96 (h) AH-6809 significantly reduced cell viability compared with the control group. **(D)** Colony formation assay showing that AH-6809 markedly decreased the number and size of colonies in Huh7 and SKHep1 cells. **(E)** Wound-healing assay showing inhibition of cell migration after 24 h of AH-6809 treatment. **(F)** Transwell invasion assay showing that AH-6809 significantly reduced the number of invasive cells in both Huh7 and SKHep1 lines. All cell-based assays (CCK-8, colony formation, wound-healing, Transwell, and Western blot) were conducted in triplicate (n = 3). Data were shown as mean ± SD. *p < 0.05, **p < 0.01, ***p < 0.001.

There was a mistake in the caption of **Supplementary Figure 3** as published. During the second round of review and revision, the authors forgot to remove the caption for **Supplementary Figure 3D**. The corrected caption of **Supplementary Figure 3** appears below:

Drug sensitivity analysis and potential small molecule targeting RARS1. (A) Drug sensitivity analysis conducted using the GSCA database revealed a strong association between RARS1 expression and resistance to various chemotherapy agents. The correlation between RARS1 and drug sensitivity is visualized through a correlation matrix showing the relationship between gene expression and chemotherapy efficacy. (B) Molecular docking analysis of AH.6809 binding to RARS1 revealed multiple binding sites, including hydrogen bonding and hydrophobic interactions. The 3D structure of RARS1 is shown with AH.6809 binding in the active site, and the detailed molecular interactions between AH.6809 and RARS1 are illustrated.

The original version of this article has been updated.

