# Supplementary figures and images for: RARS1 inhibits ENO1 ubiquitination and degradation to protect against ferroptosis in hepatocellular carcinoma
Source: Front Immunol. 2025 Dec 10;16:1686597. doi: 10.3389/fimmu.2025.1686597 (PMC12728046; doi:10.3389/fimmu.2025.1686597)

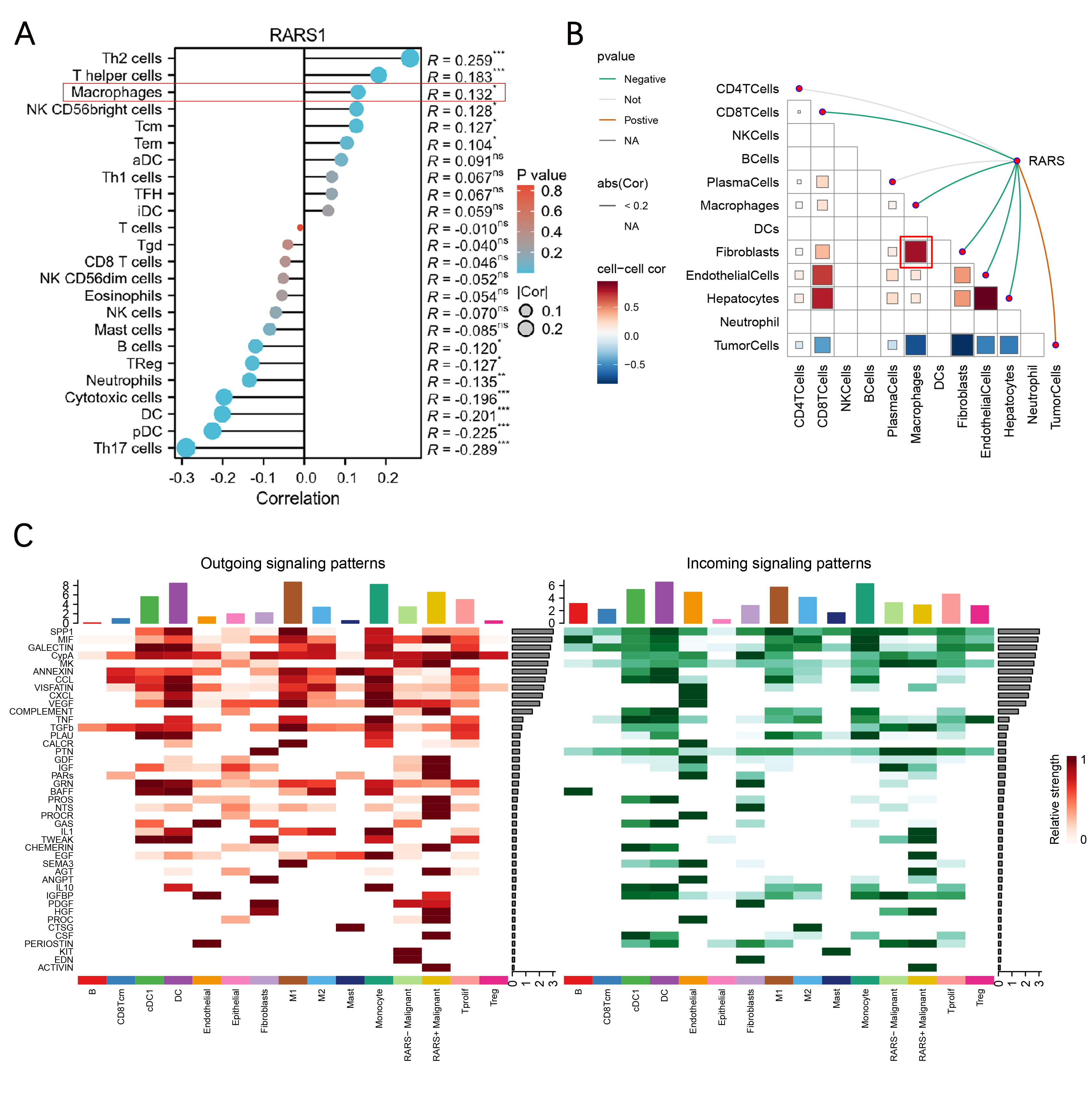

Supplement: Supplementary Figure 1 — Spatial expression of RARS1 in the tumor microenvironment. (A) RARS1 expression levels across Clusters 1–3, showing significantly higher expression in Cluster 1. (B) Spatial transcriptomic maps showing the distribution of immune and stromal cells. RARS1 expression is predominantly enriched in tumor cells (outlined in red). (C) Bar graph showing mean RARS1 expression in malignant, mixed, and normal regions. Data were shown as mean ± SD. *: p < 0.05, **: p < 0.01, ***: p < 0.001. [file Image1.jpeg]

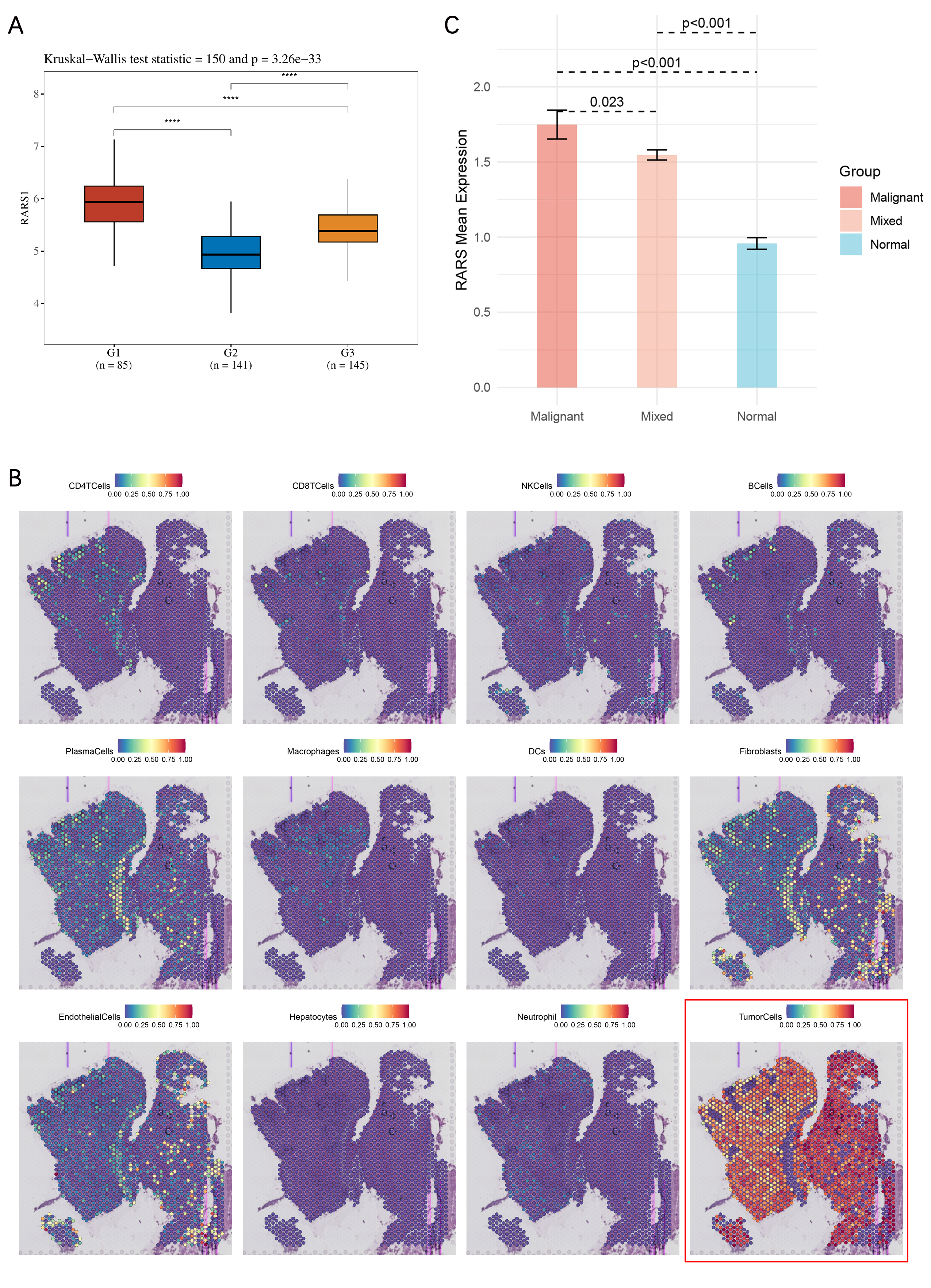

Supplement: Supplementary Figure 2 — Immune infiltration and signaling patterns in the LIHC tumor microenvironment associated with RARS1. (A) Immune cell infiltration analysis in the TCGA LIHC cohort using the ssGSEA algorithm reveals a significant positive correlation between RARS1 expression and the abundance of Th2 cells, T helper cells, and macrophages. (B) Spatial transcriptomics analysis further confirmed a strong correlation between RARS1 and the presence of macrophages and fibroblasts within the tumor microenvironment. (C) Signaling pathway analysis based on the relative strength of outgoing and incoming signaling patterns in RARS1-positive cells. The heatmaps show the relative signaling strength for various pathways, with red shades representing strong outgoing signaling and green shades representing strong incoming signaling. Data were shown as mean ± SD. *: p < 0.05, **: p < 0.01, ***: p < 0.001. [file Image2.jpeg]

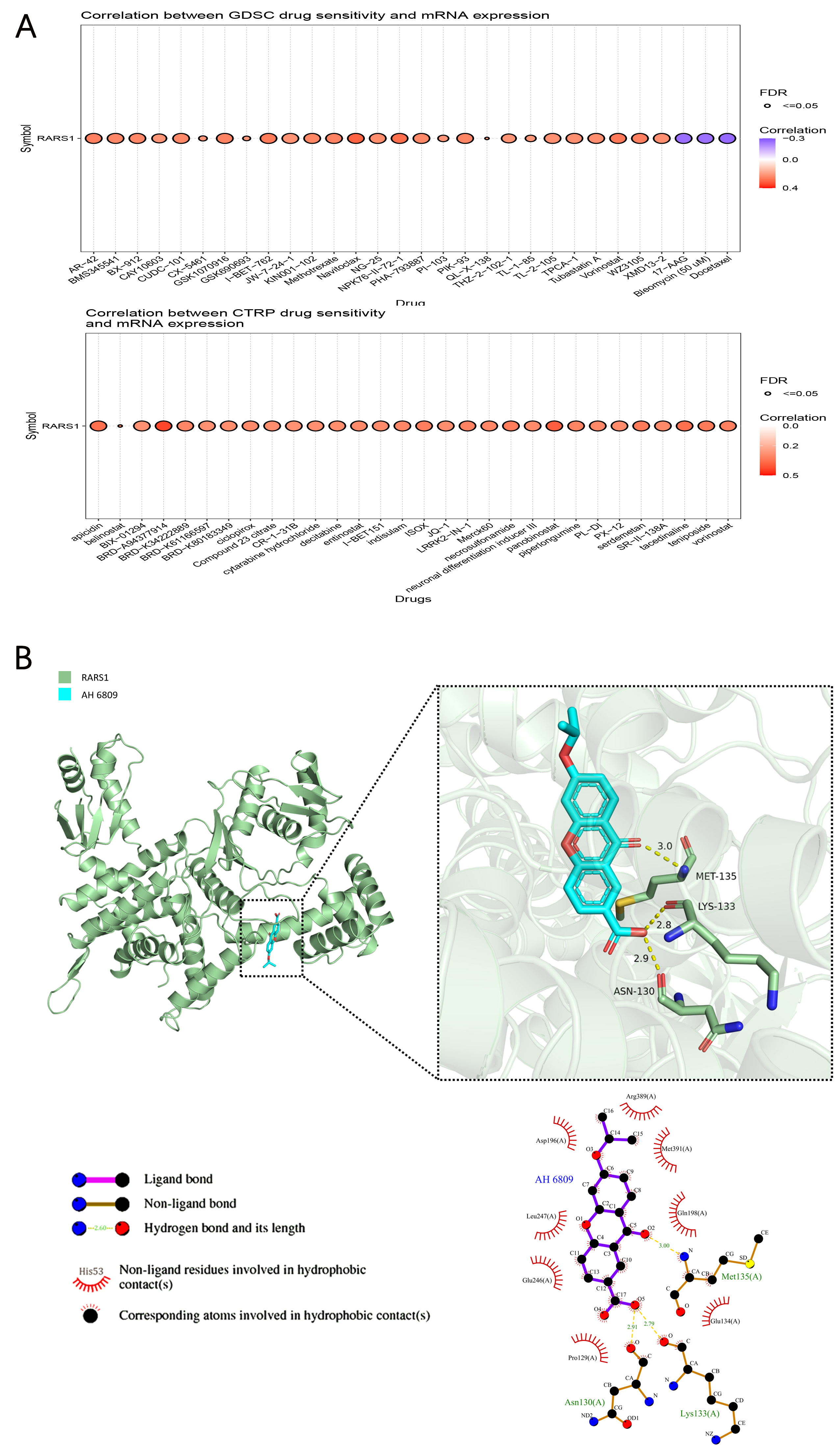

Supplement: Supplementary Figure 3 — Drug sensitivity analysis and potential small molecule targeting RARS1. (A) Drug sensitivity analysis conducted using the GSCA database revealed a strong association between RARS1 expression and resistance to various chemotherapy agents. The correlation between RARS1 and drug sensitivity is visualized through a correlation matrix showing the relationship between gene expression and chemotherapy efficacy. (B) Molecular docking analysis of AH.6809 binding to RARS1 revealed multiple binding sites, including hydrogen bonding and hydrophobic interactions. The 3D structure of RARS1 is shown with AH.6809 binding in the active site, and the detailed molecular interactions between AH.6809 and RARS1 are illustrated. [file Image3.jpeg]
