# Supplementary material for: RARS1 inhibits ENO1 ubiquitination and degradation to protect against ferroptosis in hepatocellular carcinoma
Source: Front Immunol. 2025 Dec 10;16:1686597. doi: 10.3389/fimmu.2025.1686597 (PMC12728046; doi:10.3389/fimmu.2025.1686597)
Supplement: Supplementary Table 1 — Sequences of the QRT-PCR primers used for each gene. [file Table1.docx]

| Spplemental Table 1：Sequences of the QRT-PCR primers used for each gene. | | |  |
| --- | --- | --- | --- |
| **Gene** | **Forward primer sequence** | **Reverse primer sequence** | |
| RARS1 | CAGTCTGAGGACCAGGACTC | GATGCTGGCAGGAGATGATG | |
| IL10 | GACTTTAAGGGTTACCTGGGTTG | TCACATGCGCCTTGATGTCTG | |
| IL12 | AAAATAGATGCGTGCAAGAGAGG | GGGGAAGACCTGTGACTTGAG | |
| TNF-α | CCTCTCTCTAATCAGCCCTCTG | GAGGACCTGGGAGTAGATGAG | |
| Arg1 | GTGGAAACTTGCATGGACAAC | AATCCTGGCACATCGGGAATC | |
| ACTB | CTCGCCTTTGCCGATCC | GAATCCTTCTGACCCATGCC | |
