# Supplementary material for: RARS1 inhibits ENO1 ubiquitination and degradation to protect against ferroptosis in hepatocellular carcinoma
Source: Front Immunol. 2025 Dec 10;16:1686597. doi: 10.3389/fimmu.2025.1686597 (PMC12728046; doi:10.3389/fimmu.2025.1686597)
Supplement: Supplementary Table 2 — Univariate and multivariate Cox proportional hazards regression analyses of the prognostic value of10 AR-DEGs in the TCGA LIHC cohort. [file Table2.docx]

Supplemental Table 2：Univariate and multivariate Cox proportional hazards regression analyses of the prognostic value of10 AR-DEGs in the TCGA LIHC cohort.

| Characteristics | Total(N) | HR(95% CI) Univariate analysis | *P* value Univariate analysis | HR(95% CI) Multivariate analysis | *P* value Multivariate analysis |
| --- | --- | --- | --- | --- | --- |
| IL18RAP | 373 |  |  |  |  |
| Low | 187 | Reference |  | Reference |  |
| High | 186 | 0.567 (0.398 - 0.808) | 0.002 | 0.494 (0.331 - 0.738) | < 0.001 |
| CFP | 373 |  |  |  |  |
| Low | 187 | Reference |  | Reference |  |
| High | 186 | 0.684 (0.482 - 0.970) | 0.033 | 0.834 (0.569 - 1.222) | 0.351 |
| RARS1 | 373 |  |  |  |  |
| Low | 187 | Reference |  | Reference |  |
| High | 186 | 1.952 (1.371 - 2.779) | < 0.001 | 1.567 (1.068 - 2.299) | 0.022 |
| BRCA1 | 373 |  |  |  |  |
| Low | 187 | Reference |  | Reference |  |
| High | 186 | 1.418 (1.003 - 2.005) | 0.048 | 0.945 (0.642 - 1.391) | 0.774 |
| NME1 | 373 |  |  |  |  |
| Low | 187 | Reference |  | Reference |  |
| High | 186 | 1.593 (1.125 - 2.257) | 0.009 | 1.040 (0.720 - 1.501) | 0.835 |
| ITK | 373 |  |  |  |  |
| Low | 187 | Reference |  |  |  |
| High | 186 | 0.765 (0.541 - 1.080) | 0.128 |  |  |
| CDKN2A | 373 |  |  |  |  |
| Low | 187 | Reference |  | Reference |  |
| High | 186 | 1.763 (1.243 - 2.501) | 0.001 | 1.666 (1.141 - 2.432) | 0.008 |
| CD4 | 373 |  |  |  |  |
| Low | 187 | Reference |  |  |  |
| High | 186 | 0.756 (0.535 - 1.069) | 0.114 |  |  |
| MMP9 | 373 |  |  |  |  |
| Low | 187 | Reference |  | Reference |  |
| High | 186 | 1.750 (1.231 - 2.487) | 0.002 | 1.430 (0.933 - 2.191) | 0.101 |
| CAPG | 373 |  |  |  |  |
| Low | 187 | Reference |  | Reference |  |
| High | 186 | 1.982 (1.390 - 2.826) | < 0.001 | 1.750 (1.157 - 2.648) | 0.008 |
